# Sterol Biosynthesis and Azole Tolerance Is Governed by the Opposing Actions of SrbA and the CCAAT Binding Complex

**DOI:** 10.1371/journal.ppat.1005775

**Published:** 2016-07-20

**Authors:** Fabio Gsaller, Peter Hortschansky, Takanori Furukawa, Paul D. Carr, Bharat Rash, Javier Capilla, Christoph Müller, Franz Bracher, Paul Bowyer, Hubertus Haas, Axel A. Brakhage, Michael J. Bromley

**Affiliations:** 1 Manchester Fungal Infection Group, Institute of Inflammation and Repair, University of Manchester, Manchester, United Kingdom; 2 Department of Molecular and Applied Microbiology, Leibniz Institute for Natural Product Research and Infection Biology (HKI), Jena, Germany; 3 Microbiology Unit, Medical School, Universitat Rovira i Virgili, Reus, Spain; 4 Department of Pharmacy, Center for Drug Research, Ludwig-Maximilians University of Munich, Munich, Germany; 5 Division of Molecular Biology, Biocentre, Medical University of Innsbruck, Innsbruck, Austria; 6 Institute for Microbiology, Friedrich Schiller University Jena, Jena, Germany; University of Rochester, UNITED STATES

## Abstract

Azole drugs selectively target fungal sterol biosynthesis and are critical to our antifungal therapeutic arsenal. However, resistance to this class of drugs, particularly in the major human mould pathogen *Aspergillus fumigatus*, is emerging and reaching levels that have prompted some to suggest that there is a realistic probability that they will be lost for clinical use. The dominating class of pan-azole resistant isolates is characterized by the presence of a tandem repeat of at least 34 bases (TR34) within the promoter of *cyp51A*, the gene encoding the azole drug target sterol C14-demethylase. Here we demonstrate that the repeat sequence in TR34 is bound by both the sterol regulatory element binding protein (SREBP) SrbA, and the CCAAT binding complex (CBC). We show that the CBC acts complementary to SrbA as a negative regulator of ergosterol biosynthesis and show that lack of CBC activity results in increased sterol levels via transcriptional derepression of multiple ergosterol biosynthetic genes including those coding for HMG-CoA-synthase, HMG-CoA-reductase and sterol C14-demethylase. In agreement with these findings, inactivation of the CBC increased tolerance to different classes of drugs targeting ergosterol biosynthesis including the azoles, allylamines (terbinafine) and statins (simvastatin). We reveal that a clinically relevant mutation in HapE (P88L) significantly impairs the binding affinity of the CBC to its target site. We identify that the mechanism underpinning TR34 driven overexpression of *cyp51A* results from duplication of SrbA but not CBC binding sites and show that deletion of the 34 *mer* results in lack of *cyp51A* expression and increased azole susceptibility similar to a *cyp51A* null mutant. Finally we show that strains lacking a functional CBC are severely attenuated for pathogenicity in a pulmonary and systemic model of aspergillosis.

## Author Summary

*Aspergillus fumigatus* is the most important airborne mould pathogen and allergen worldwide. Estimates suggest that >3 million people have invasive or chronic infections that lead to >600,000 deaths every year. Very few drugs are available to treat the various forms of aspergillosis and we rely predominantly on the azole class of agents which inhibit sterol biosynthesis. Resistance to the azoles is growing alarmingly, primarily driven by strains with two principal genetic signatures (TR_34_/L98H and TR_46_/Y121F/T289A). In this study we identify that the transcriptional mechanism governing resistance in this group of isolates is linked to the opposing actions of 2 transcriptional regulators, SrbA and the CBC, and uncover a role for the CBC in sterol regulation and virulence in *A*. *fumigatus*. We propose targeting SrbA would provide an effective avenue for therapeutic intervention for resistant strains.

## Introduction

Sterols are components of most eukaryotic cell membranes playing key roles in sustaining membrane integrity and fluidity. The *de novo* synthesis of sterols by fungi is essential for their viability and numerous antifungal drugs have been developed that exploit the differences between enzymes in the sterol biosynthetic pathway of fungal pathogens and their hosts. The most notable sterol biosynthetic inhibitors are the azoles which are extensively used in crop protection and have been the cornerstone of systemic antifungal therapy in man for the last 30 years [1].

Triazoles such as voriconazole (VORI), itraconazole (ITRA) or posaconazole (POSA) represent the main antifungal drug class employed to treat disease caused by *Aspergillus spp*. Although in general *Aspergillus fumigatus* is susceptible to these drugs, resistance is emerging and reaching levels that have prompted some health centres to move away from the use of azoles as a sole first line therapeutic, opting instead for high cost combination therapies and/or less effective agents [2,3,4,5]. Azole drugs act by inhibiting the function of the sterol C14-demethylase Cyp51, leading to ergosterol depletion and simultaneous accumulation of toxic sterol compounds [6]. A principal cause of azole resistance in clinical strains of *A*. *fumigatus* is modification of the *cyp51A* gene, one of two genes that encode isoforms of sterol C14-demethylase in this pathogen. A particular family of pan-azole resistant isolates dominates. Typified by the TR34/L98H modification, but also including TR46/Y121F/T289A and TR53, they harbor a tandem repeat in the promoter of *cyp51A* along with a non-synonymous mutation resulting in one or more amino acid changes in the Cyp51A protein. In the case of TR34/L98H this is manifest as a duplication of a 34 *mer* within the 5’ non-translated (5’ NTR) region of *cyp51A*, combined with a lysine to histidine substitution at position 98 in the protein. Understanding the mechanisms by which the TR34/L98H family and non-*cyp51A* type mutations lead to resistance is critical to formulating strategies to both detect and treat resistant infections.

What drives resistance in the TR34/L98H family is only partially understood. Introduction of the L98H modification into a hitherto wild-type (wt) isolate of *A*. *fumigatus* results in a modest but significant increase in triazole tolerance. Introduction of the TR34 variant into a conventional *cyp51A* promoter results in an approximate doubling of *cyp51A* gene expression and an associated increase in tolerance to azoles [7]. Only when these modifications are combined can isolates reach tolerance levels that exceed clinically relevant breakpoints as defined by EUCAST (European Committee on Antimicrobial Susceptibility Testing) [8]. Although the mechanistic basis of L98H driven increase in azole tolerance has been linked to modification of the tertiary structure of Cyp51A [7], the cause of increased expression resulting from the TR34 promoter variant has not been elucidated.

In mammalian systems, sterol production is transcriptionally regulated by sterol regulatory element binding proteins (SREBPs). SREBPs belong to the basic helix-loop-helix (bHLH) transcription factors. In their inactive form they are membrane-bound, once activated the regulator is released and accumulates in the nucleus where it binds to sterol regulatory elements (SREs) in the promoters of target genes, including sterol C14-demethylase and activates expression [9,10]. At sterol excess SREBPs remain inactive causing decreased transcript levels of their targets [10]. The activating role of the SREBPs is facilitated by the action of the CCAAT-binding complex (CBC) NF-Y which is made up of three subunits NF-YA, NF-YB and NF-YC. The SREBPs and NF-Y synergistically activate expression of essentially all genes involved in sterol metabolism [11].

Orthologues to mammalian SREBPs have been found in several fungi including *A*. *fumigatus*. The *A*. *fumigatus* SREBP homologue, termed SrbA, is a non-redundant transcription factor which, like its mammalian counterparts, plays a key role in the regulation of sterol biosynthetic genes, including *cyp51A*. In keeping with this role, *A*. *fumigatus* strains lacking SrbA have reduced tolerance to the azoles [12,13,14].

Similarly, *A*. *fumigatus* has an orthologue of the CBC. As with the mammalian regulator, the *A*. *fumigatus* CBC is a multimeric transcription factor complex comprising three subunits (HapB/HapC/HapE) and is highly conserved from yeast (Hap2p/Hap3p/Hap5p) to man (NF-YA/NF-YB/NF-YC) [11,15,16,17,18,19]. A mutation in the HapE subunit (P88L) was recently identified as the causative modification that led to azole resistance in an *A*. *fumigatus* strain isolated from a patient in the Netherlands [20].

As both SrbA and the CBC have been directly implicated in modified azole tolerance in *A*. *fumigatus*, and their orthologues have positively acting sterol regulatory functions, we sought to understand the role of the CBC in sterol biosynthesis and both regulators in clinical azole resistance. Particularly we were interested in uncovering any role of these regulators in TR34 mediated resistance.

## Results

### The CBC recognises a CGAAT motif within the azole resistance associated 34 mer in the promoter of cyp51A, a key gene in the sterol biosynthetic pathway

**Fig 1:**
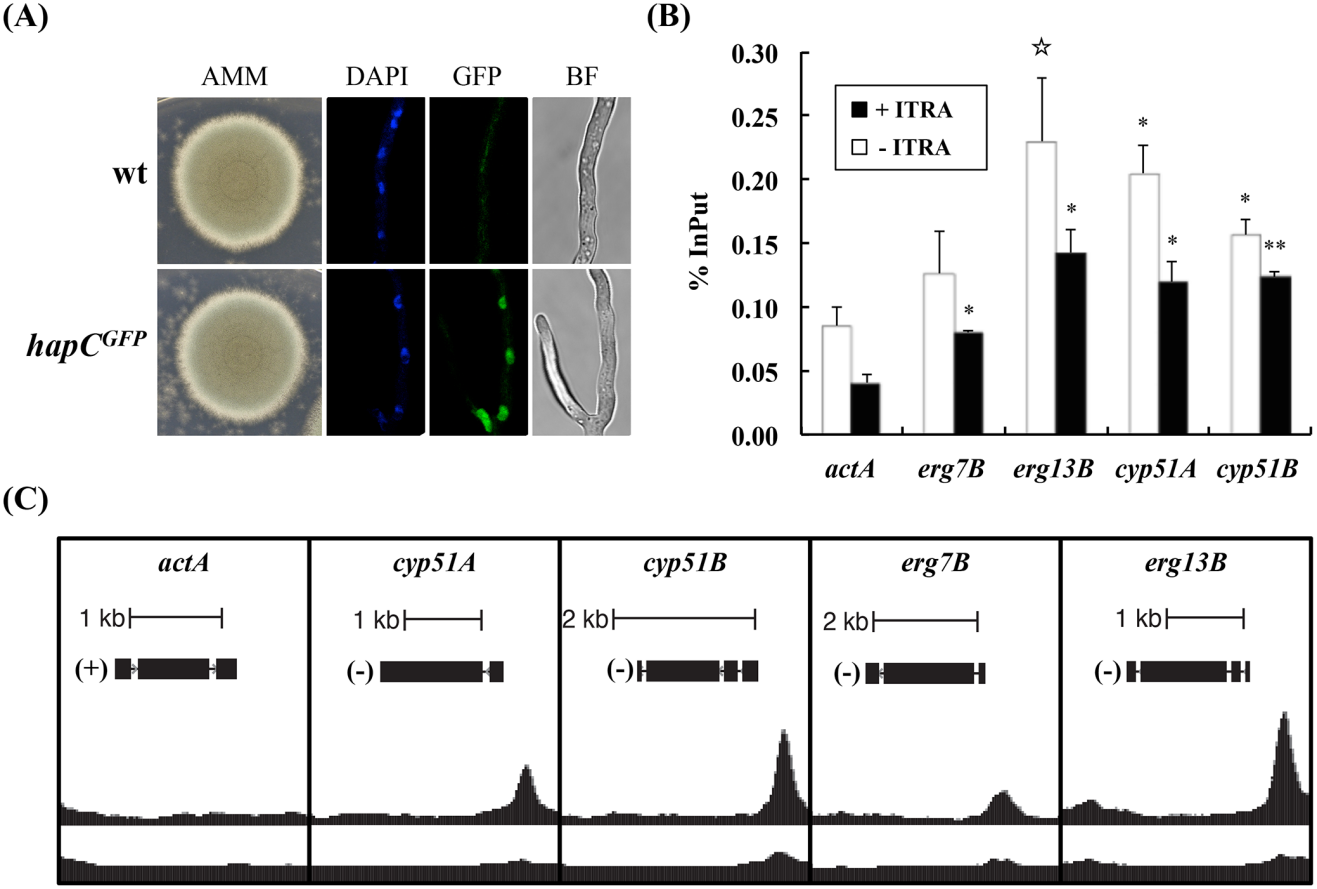
The CBC is a direct regulator of ergosterol biosynthetic genes. (A) The *hapC* ^*GFP*^ strain is phenotypically indistinct from the wt host and HapC^GFP^ localises to the nucleus. For confocal laser microscopy 10^4^ spores of the respective isolates were grown in liquid AMM at 37°C for 24 h. DAPI was used to co-stain the nuclei. (B) *In vivo* binding of the CBC was confirmed by comparing % recovery of DNA (ChIP-qPCR) from promoters of ergosterol biosynthetic genes (*erg7B*, *erg13B*, *cyp51A* and *cyp51B*) to an unbound region of the *actA* promoter (for more details, see Material and Methods). Samples have been assessed in duplicate. Error bars indicate the standard deviation of respective samples and p-values were calculated by Student’s T-test (reference: *actA*): ☆, <0.06; *, <0.05; ** <0.01. (C) Highlighted ChIP-seq peaks at promoters of genes that were validated by ChIP-qPCR. For ChIP experiments liquid cultures were grown for 18 h at 37°C in AMM.

**Fig 2:**
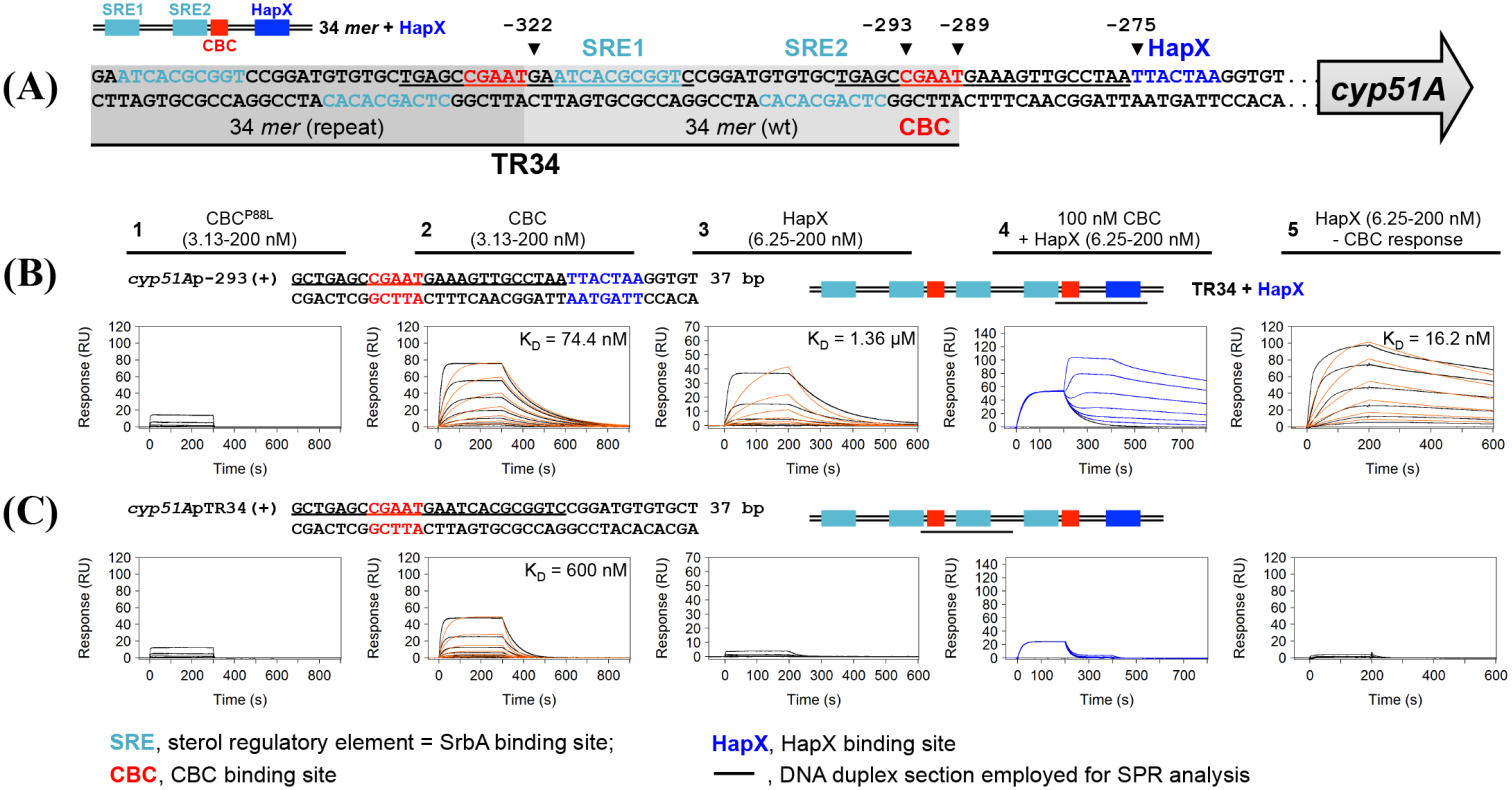
The CBC binds to a CGAAT motif within the 34 mer of the cyp51A promoter, which is facilitated HapX. This interaction is perturbed by the HapE^P88L^ modification. (A) Schematic depiction of the *cyp51A* TR34 promoter region. (B) & (C) To further analyse the *in vivo* binding site for the CBC *in vitro*, real-time SPR analysis was employed. Binding of the mutated CBC^P88L^ (HapB/HapC/HapE^P88L^) to DNA (panel 1), wt CBC (HapB/HapC/HapE) to DNA (panel 2), HapX to DNA (panel 3) and HapX to preformed CBC/DNA complexes (panel 4) was monitored. The SPR sensorgrams are shown from sensor-immobilised 37 base pair DNA dduplexes containing the CGAAT motifs of the respective wt promoter section (*cyp51A*-293) as well as the TR34 tandem repeat variant (*cyp51A*-TR34). Underlined nucleotides are covered upon CBC binding according to the CBC/DNA binary complex crystal structure [23]. Binding responses of the indicated CBC or HapX concentrations injected in duplicate (black lines) are shown overlaid with the best fit derived from a 1:1 interaction model including a mass transport term (red lines). Sensorgrams in panel 5 depict the association/dissociation responses of HapX on preformed CBC/DNA and were generated by CBC response (co-injection of buffer instead of HapX) subtraction from HapX co-injection responses. Dissociation constants (K_D_) are given inside the graphs.

The *A*. *fumigatus* CBC functions as a heterotrimer comprising HapB, HapC and HapE and is known to interact with the highly abundant consensus motifs CCAAT and CGAAT [21,22]. A number of potential binding sites are evident within the *cyp51A* promoter, therefore we sought to identify if the CBC interacted directly with the promoter of *cyp51A* using chromatin-immunoprecipitation followed by next-generation sequencing (ChIP-seq). To this end, we replaced the native *hapC* gene in isolate A1160P+ with a chimeric *gfp*-construct. The respective strain expresses C-terminally GFP-tagged HapC protein (*hapC* ^*GFP*^, Fig 1A). We sequenced anti-GFP bound DNA fractions from cell extracts and identified a single region within the promoter (within 1kb upstream of the translational start site) of *cyp51A* to which the GFP-CBC was bound (S1 Table). The sequencing peak identified by ChIP-seq was at position -297 relative to the translation start placing it at the 3’ end of the 34 *mer*. This result was validated by ChIP-qPCR by comparing enrichment of the region identified in the ChIP-seq analysis with region not bound by the CBC in the promoter of *actA* (Fig 1B). By examining the promoter region around the ChIP-seq peak, we found the CBC binding motif CGAAT at position -293 to -289 (Fig 2A). To further analyse the interaction of the CBC to this motif, binding kinetics and affinity were monitored by surface plasmon resonance spectroscopy (SPR) protein-DNA interaction analysis. Kinetic CBC binding responses to a 37-bp *cyp51A* promoter duplex revealed a dissociation constant (K_D_) of 74.4 nM, demonstrating that the CBC effectively recognised the CGAAT^-293^ motif within the original 34 *mer in vitro* (Fig 2B, upper panel 2). Importantly, although the CGAAT sequence is duplicated in TR34 (CGAAT^-327^), the binding affinity of the CBC at the duplicated sequence is about 8-fold decreased and consequently not effectively bound (Fig 2C panel 2).

### HapX, a transcriptional regulator associated with adaptation to environmental iron cues, binds to the promoter of cyp51A

Cyp51A is known to use iron as a catalytic co-factor and the CBC is a known interaction partner of HapX, an iron responsive basic region leucine zipper (bZIP) transcription factor. We therefore screened the nucleotide sequence downstream the CGAAT^-293^ motif for potential HapX binding sites [19,21,24]. We identified a pseudo-palindromic TTACTAA sequence at position -275 to -269 in the *cyp51A* promoter that corresponds exactly to the yeast AP-1 (YAP1) bZIP consensus binding site [25]. Employing SPR co-injection analysis we confirmed that this region of the *cyp51A* promoter is synergistically bound by the CBC/HapX complex (Fig 2B panel 4 and 5). Consistent with the fact that the HapX-binding site is not duplicated in the 34 base tandem repeat, combinatorial CBC/HapX recognition was not detectable on a DNA duplex that contained the duplicated CGAAT motif (Fig 2C, panel 4 and 5).

Together these data demonstrate that only the wt 34 *mer* of the *cyp51A* promoter is a direct target of the CBC and its interaction partner HapX.

### The CBC represses cyp51A expression, this repression is liberated in a HapE^P88L^ mutant

**Fig 3:**
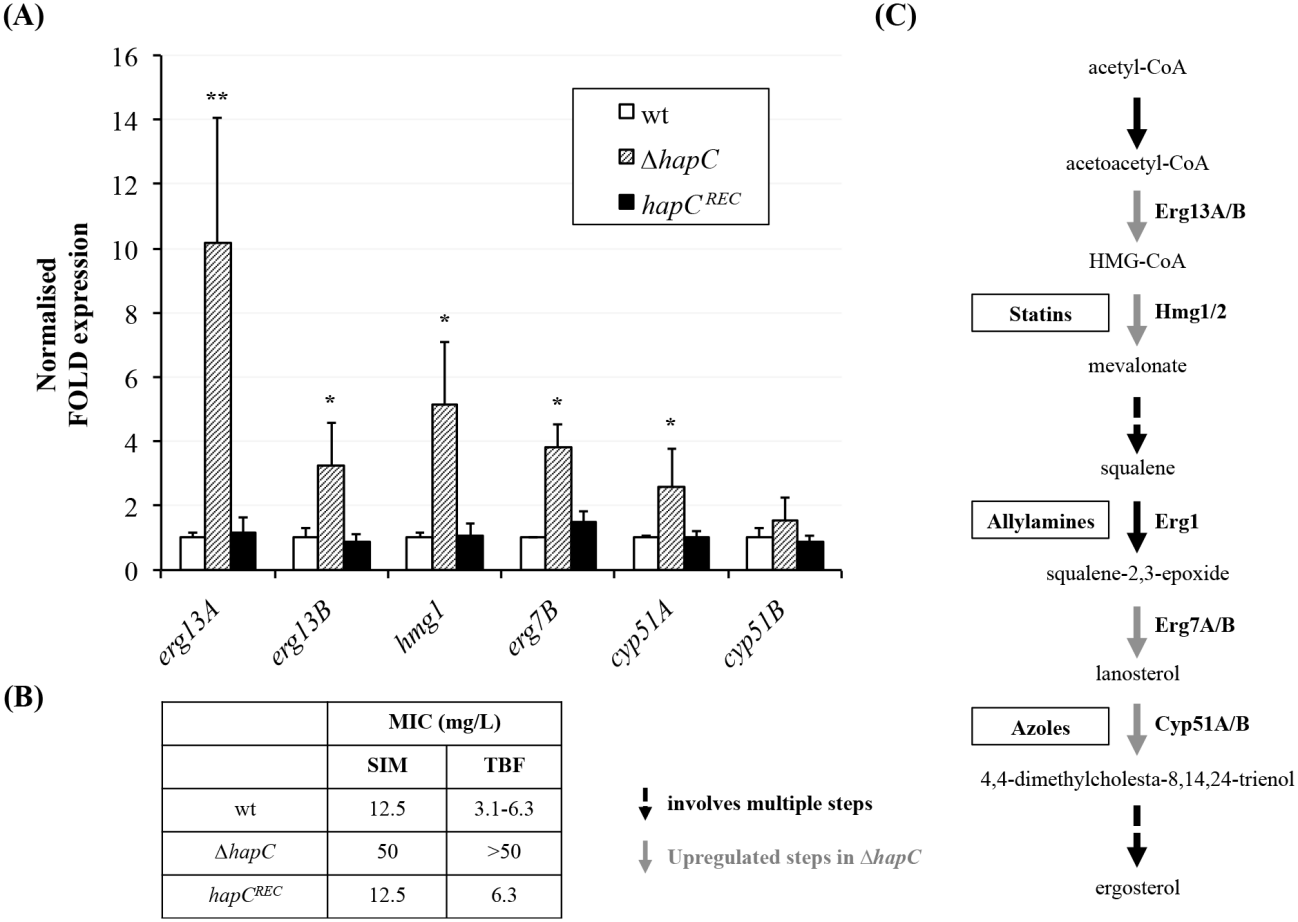
*hapC* deletion leads to derepression of ergosterol biosynthetic genes and confers resistance to non-azole ergosterol biosynthesis inhibitors. (A) Transcript levels of six ergosterol biosynthetic genes have been monitored in Δ*hapC* using RT-qPCR (*gpdA* was used as reference gene). Strains were grown in AMM for 18h at 37°C, 200 rpm. Results represent the mean of biological triplicates and error bars illustrate the standard deviation. p-values were calculated by Student’s T-test (reference: wt): *, <0.05; ** <0.01. (B) MIC levels of simvastatin and terbinafine, both blocking enzymes upstream Cyp51. (C) Schematic of ergosterol biosynthetic pathway including targets for inhibitors.

To determine if the CBC is functioning as a repressor, or an activator of *cyp51A* gene expression and to assess the role of the HapE^P88L^ mutation we generated CBC gene deletion mutants of each subunit of the CBC namely, HapB (Δ*hapB*), HapC (Δ*hapC*) and HapE (Δ*hapE*), a strain expressing HapE^P88L^ (*hapE* ^*P88L*^), and a deletion mutant of HapX (Δ*hapX*) (S2 Table). Disruption of any of the CBC subunits, HapX or mutation of HapE resulted in increased tolerance to ITRA, VORI and POSA (Table 1). Additionally, similar to HapE^P88L^ [20] *cyp51A* expression was increased by about 2-fold in the Δ*hapC* strain (Fig 3A). This result indicates that the CBC and HapX act as repressors of *cyp51A* and that the HapE^P88L^ mutation abolishes this repressing function. The loss of repressing activity of HapB/HapC/HapE^P88L^ seems to be linked to a reduction in binding affinity of the mutated CBC to the CGAAT^-293^ motif as indicated by SPR analysis (Fig 2B; compare panel 1 to panel 2).

**Table 1:** Azole sensitivity of strains generated in this study. MIC testing has been carried out using the EUCAST broth microdilution reference method [26].

	MIC (mg/L)		
	ITRA	VORI	POSA
Wt	0.5	0.5	0.03–0.06
Δ*hapC*	>16	2	0.5
*hapC* ^*REC*^	0.5	0.5	0.06
Δ*hapB*	>16	2	0.5
Δ*hapE*	>16	2	0.5
Δ*cyp51A*	0.13	0.25	0.016
Δ*hapC*Δ*cyp51A*	0.25	0.5	0.016–0.03
*hapE* ^*P88L*^	16	2	0.5
Δ*hapX*	1	1	0.25

Combined, these results suggest that azole resistance caused by HapE^P88L^ is linked to impaired CBC DNA-binding function.

### The CBC is a repressor of several genes in the ergosterol biosynthetic pathway

The discrepancy between the increase in expression of *cyp51A* (c. 2-fold) and azole resistance (>32-fold) suggested that *cyp51A*-independent factors may be influencing azole resistance in CBC deficient strains. This is supported by the fact that a Δ*hapCΔcyp51A* is more resistant to the azoles than a single *cyp51A* null mutant (Table 1). We therefore hypothesised a possible function of the CBC in the repression of other genes in the ergosterol biosynthetic pathway.

Interrogation of our ChIP-seq data allowed us to identify CBC binding sites in the promoter regions (< 1.0 kb 5’ NTR) of around half of the ergosterol biosynthetic genes (14 out of 27, S1 Table). We validated the results of the ChIP-seq analysis by ChIP-qPCR for four of the genes *erg13B*, *erg7B*, *cyp51A* and *cyp51B* (Fig 1B). To assess if binding of the CBC was affected by azole treatment, which would mimic cellular sterol depletion, we performed ChIP-qPCR using *A*. *fumigatus* cultures grown in the presence or absence of ITRA. We found enrichment of the binding regions in both sterol replete (-ITRA) and sterol deplete (+ITRA) conditions indicating that the CBC is constitutively bound to all four promoters under these conditions (Fig 1B).

To investigate if binding of the CBC was linked to regulation of gene expression, we monitored expression levels of several genes associated with the ergosterol biosynthetic pathway in our CBC mutant (Δ*hapC*). Specifically we monitored genes coding for enzymes of the first and committed steps in ergosterol biosynthesis, HMG-CoA synthase (paralogs *erg13A* and *erg13B*) and HMG-CoA reductase (paralogs *hmg1 and hmg2*), the genes encoding the azole drug target sterol C14-demethylase (paralogs *cyp51A* and *cyp51B*) as well as *erg7B*, that encodes lanosterol synthase. For four of these genes, *erg13A*, *erg13B*, *hmg1* and *erg7B*, expression was significantly increased by at least 3-fold in Δ*hapC* when compared to the isogenic host strain (Fig 3A). For *cyp51B* we detected a modest increase in transcript levels (>1.5 fold). We were unable to detect expression of *hmg2* in either strain due to the very low abundance of the transcript. It is noteworthy that our ChIP-seq results did not indicate binding of the CBC to the 1.5 kb region upstream of the *erg13A* translation start site, suggesting an indirect regulation of this gene by the CBC.

Based on the idea that increased expression of several ergosterol biosynthetic genes in CBC mutants might lead to increased activity of the enzymes, we determined MIC levels of Δ*hapC* for simvastatin and terbinafine (Fig 3B). These drugs, belonging to the families of statins and allylamines, are ergosterol biosynthesis inhibitors that inhibit the enzymes HMG-CoA reductase (Hmg) and squalene epoxidase (Erg1) respectively. Both of these enzymes perform functions upstream of the C14-demethylation carried out by the Cyp51 enzymes (Fig 3C). In keeping with our hypothesis, Δ*hapC* displayed a significant increase in tolerance to both of these drugs.

Taken together, these data illustrate that loss of the CBC leads to upregulation of genes involved in several steps of ergosterol biosynthesis, and increases the activity of several enzymatic steps in this pathway. This results in resistance to ergosterol biosynthesis inhibitors belonging to the azoles, statins and allylamine drug classes.

### CBC mutation results in elevated sterol levels

Resistance to simvastatin, terbinafine and azole drugs suggests that CBC mutation leads to increased activity of the whole ergosterol biosynthetic pathway. To analyse the effect of loss of CBC function on ergosterol biosynthesis, sterol levels were quantified using GC-MS. To confirm the validity of our results we analysed the ergosterol levels in a *srbA* null mutant (Δ*srbA*). Loss of SrbA function is documented to result in decreased ergosterol levels and increased C4-methylated sterols (Table 2, columns 6 and 7) [14]. In keeping with this we demonstrated that ergosterol levels were decreased (Table 2, column 2: 3.13-fold) in Δ*srbA* and an accumulation of eburicol (Table 2, column 6:, 2.37-fold) and 4,4-dimethylergosta-8,24(28)-dien-3β-ol (Table 2, column 7: 11.03-fold) was observed. In line with our expectations, Δ*hapC* showed a 2.4-fold increase in ergosterol levels (Table 2, column 2). In addition to ergosterol, which constitutes around 90% of the total sterol content in Δ*hapC*, we found four further sterols to be elevated in this mutant, including the Cyp51 substrate molecules lanosterol (Table 2, column 5: 1.67-fold) and eburicol (Table 2, column 6: 2.09-fold). The Δ*cyp51A*Δ*hapC* isolate displayed increased levels of ergosterol (Table 2, column 2: 1.59-fold) but also ergosta-5,7,24(28)-trien-3β-ol (Table 2, column 3: 1.65-fold) and episterol (Table 2, column 4: 2.51-fold). The *hapE* ^*P88L*^ strain showed ergosterol levels similar to that of the wt however, ergosta-5,7,24(28)-trien-3β-ol (Table 2, column 3: 3.57-fold) and episterol (Table 2, column 3: 3.57-fold) were increased in this mutant indicating that the regulatory defect in this mutant differs somewhat from the null mutant.

**Table 2:** CBC mutants show increased production of sterols. Sterol levels of transcription factor mutants incubated in AMM for 24 h at 37 C, 200 rpm have been determined by GC-MS. Δ*cyp51A* served as control for the double deletion mutant Δ*hapC*Δ*cyp51A*. Sterol content has been normalised to that of wt. Samples were assessed in biological triplicates. p-values were calculated by Student’s T-test (reference: wt): *, <0.05; ** <0.01. TS, total sterols; bold, >1.5 fold increase.

	Sterol content (FOLD)							
	1	2	3	4	5	6	7	TS
**wt**	1.00	1.00	1.00	1.00	1.00	1.00	1.00	1.00
**Δ*hapC***	1.08	2.40*	3.99**	4.84*	1.67*	2.09*	0.98	2.36*
***hapC*** ^***REC***^	0.96	1.01	1.16	1.01	0.90	1.07	0.76	1.00
***hapE*** ^***P88L***^	0.98	1.04	3.57**	2.36*	0.73	0.77	1.06	1.07
**Δ*cyp51A***	0.81	0.90	1.30	1.22	1.16	0.63	**1.50**	0.91
**Δ*cyp51A*Δ*hapC***	1.03	1.59*	1.65*	2.51*	0.70	1.40	0.35	1.54*
**Δ*srbA***	0.64	0.32	0.00	0.00	0.52	2.37*	11.03*	0.47

**1**, ergosta-5,7,9(11),22-tetraen-3β-ol; **2**, ergosterol; **3**, ergosta-5,7,24(28)-trien-3β-ol; **4**, episterol; **5**, lanosterol; **6**, eburicol; **7**, 4,4-dimethylergosta-8,24(28)-dien-3β-ol.

These findings suggest that transcriptional upregulation of ergosterol biosynthetic genes in Δ*hapC* leads to increased sterol levels, particularly ergosterol, which is likely to contribute to resistance to ergosterol biosynthesis inhibitors in CBC mutants.

### CBC mutation results in severe growth reduction and attenuated virulence in a murine model of aspergillosis

**Fig 4:**
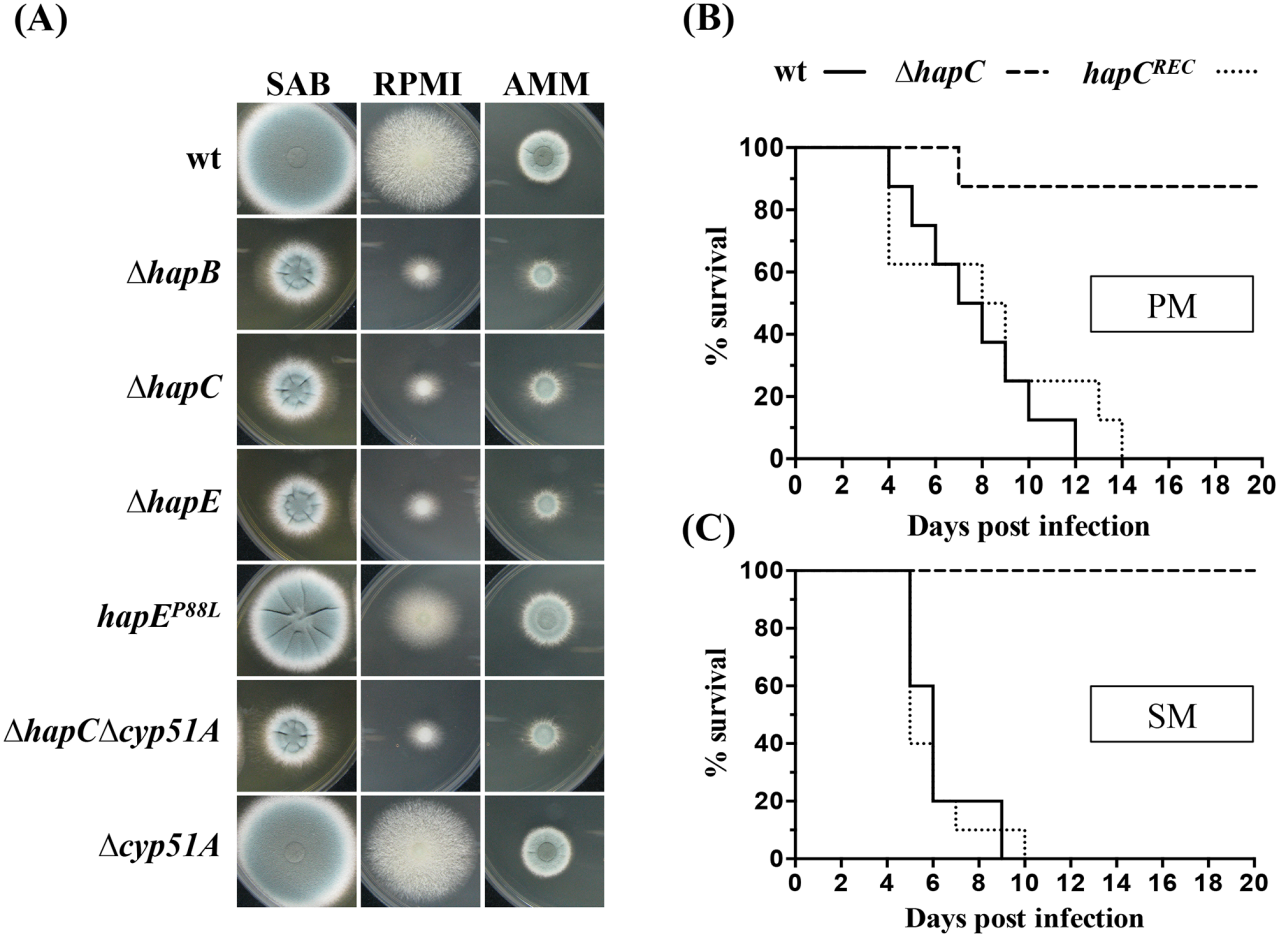
The CBC plays a crucial role in the growth of *A*. *fumigatus* and is required for pathogenicity in a pulmonary (non-leucopenic) and systemic (leucopenic) model of aspergillosis. (A) Radial growth of strains was determined under different nutrient supply. Strains were grown on AMM, RPMI as well as SAB solid medium; (B) Survival of cortisone acetate immunosuppressed OF-1 mice challenged intranasally with 1x10^5^ CFU/animal of *A*. *fumigatus*. P < 0.05 in comparison to wt and *hapC* ^*REC*^. PM, pulmonary model; (C) Survival proportions of cyclophosphamide immunosuppressed OF-1 mice challenged intravenously with 3x10^4^ CFU/animal of *A*. *fumigatus*. P < 0.05 in comparison to wt and *hapC* ^*REC*^. SM, systemic model.

The high abundance of CCAAT and CGAAT motifs in eukaryotic promoters suggests that the expression of a large proportion of protein coding genes might be affected by CBC mutation. Hence, it seemed likely that CBC defective mutants would have significant phenotypic defects. We assessed growth of CBC mutants (Δ*hapB*, Δ*hapC*, Δ*hapE*, *hapE* ^*P88L*^) on *Aspergillus* minimal medium (AMM), RPMI as well as Sabouraud dextrose agar (SAB, rich-nutrient medium). Lack of either subunit resulted in significant growth defects on all media with individual deletion mutants showing identical phenotypes (Fig 4A). The *hapE* ^*P88L*^ isolate revealed a less dramatic phenotype compared to *hap*-deletion mutants. Growth of this isolate, carrying a single amino acid mutation, was impaired particularly on RPMI.

To assess the requirement of the CBC in infection, we compared the virulence of Δ*hapC* and *hapC* ^*REC*^ with that of the isogenic control strain in systemic and pulmonary murine models of aspergillosis. The pathogenicity of the isogenic control and *hapC* ^*REC*^ strains were indistinguishable, causing 100% mortality (n = 10) between 9 and 10 days after systemic infection and between 12 and 14 days after pulmonary infection. Systemic infection by Δ*hapC* caused no mortality while in the pulmonary model only one animal succumbed to the infection (Fig 4B). Tissue burden studies showed a reduced fungal load in animals infected i.v. (n = 10) and i.n. (n = 8) with Δ*hapC* in comparison to those challenged with wt and *hapC* ^*REC*^ (S1 Fig). Pulmonary infection resulted in high fungal burden in all animals challenged with wt and *hapC* ^*REC*^ while Δ*hapC* was cleared in all mice except two. Kidneys from animals inoculated with wt or *hapC* ^*REC*^ showed low viable fungal elements, however no colonies were detected in Δ*hapC-*infected mice. With exception of the brain, the systemic infection resulted in high fungal loads in all tissues after wt and *hapC* ^*REC*^ inoculation. Animals challenged with Δ*hapC* showed significantly lower CFU/g in all organs except for the liver and brain when compared to those challenged with wt or *hapC* ^*REC*^.

Taken together, these results demonstrate the crucial role of the CBC for *A*. *fumigatus* growth and show that transcriptional circuits mediated by this regulator are critical during infection of an immunocompromised host.

### The positive regulator of transcription SrbA binds to the 34 mer in the cyp51A promoter

Our analysis of the CBC suggests that it is associated with the 34 *mer* but not directly responsible for the transcriptional enhancement of *cyp51A* observed in strains with the TR34 mutation. This led us to assess if the other known transcriptional regulator of sterol biosynthetic genes, SrbA, was involved.

**Fig 5:**
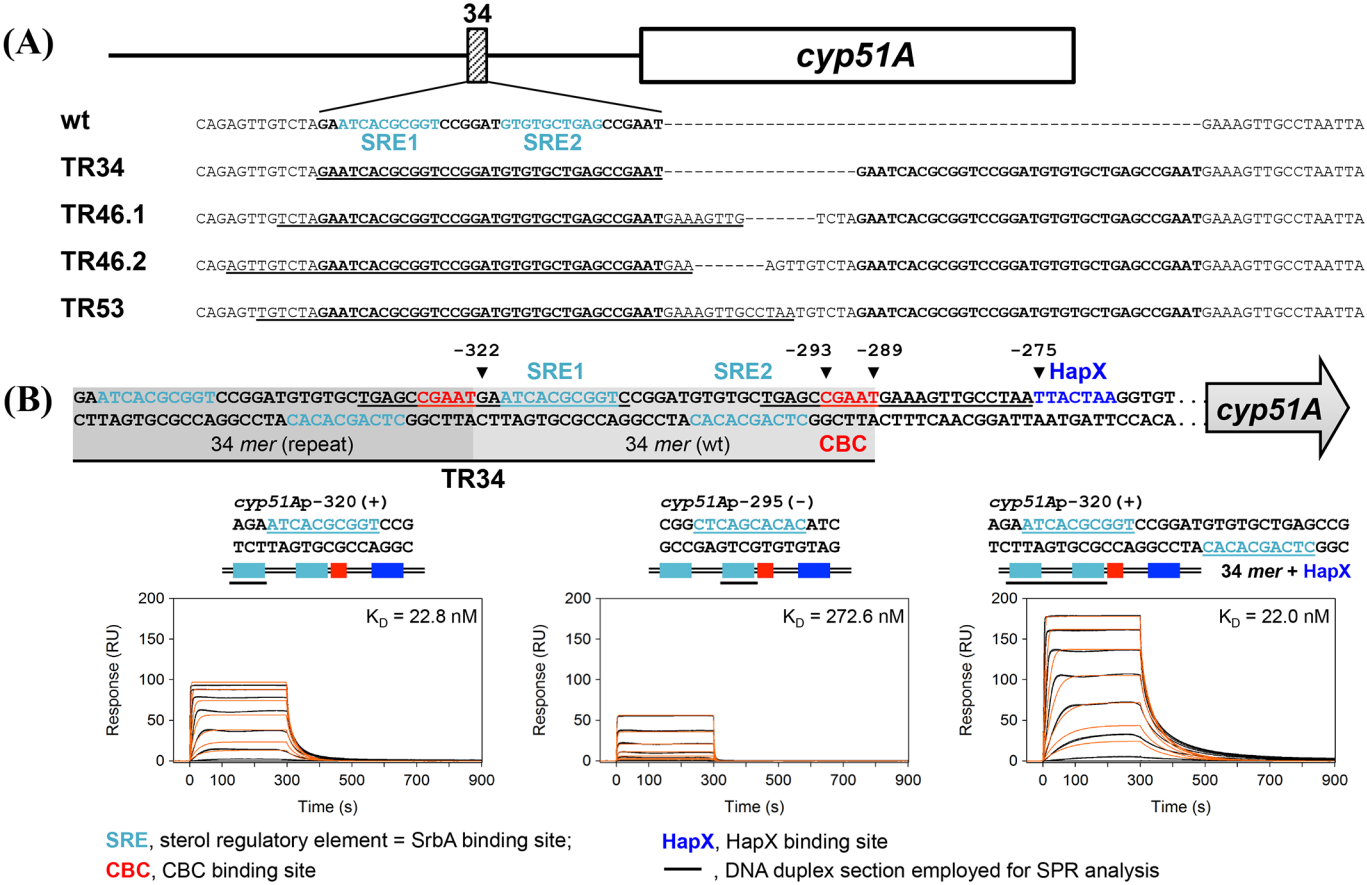
SrbA interacts with a transcriptional enhancer within the 34 *mer*. (A) Alignment of TR34, TR46.1, TR46.2 and TR53. The 34 *mer* is duplicated in all TR variants (adapted from [30]). bold, 34 *mer*; underlined, duplicated sequence. (B) To further analyze the *in vivo* binding data described by Chung *et al*. [27], we measured binding of recombinant SrbA to this section using real-time SPR analysis. The putative binding sites identified by comparison to the consensus sequence are shown as SRE1 and SRE2. DNA duplexes containing either SRE1, SRE2 or both were used for SPR analysis. Sensorgrams of 200, 100, 50, 25, 12.5, 6.25, and 3.13 nM SrbA161-267 binding injected in duplicate (black lines) are shown overlaid with the best fit derived from a 1:1 interaction model including a mass transport term (red lines).

In *A*. *fumigatus* the sterol regulatory element binding protein SrbA has been described as direct positive regulator of sterol biosynthesis in general and *cyp51A* in particular [13,27]. A previous study identified a motif similar to the reported sterol regulatory element (Sre1) binding motif of *S*. *pombe* upstream of the TR34 site [13]. In an attempt to corroborate the location of this site, we performed a retrospective analysis of a recent genome-wide ChIP-Seq evaluation of SrbA binding [27] and identified a sequencing peak summit at position -302 from the *cyp51A* coding sequence start site, which in contrast to the previous predicted location of the binding site, is within the duplicated 34 *mer*. By comparing the 34 bases of TR34 with the SrbA consensus binding motif [27] we identified two putative SREs (SRE1 and SRE2, Fig 5A). These SREs are also evident in the overlapping TR46 and TR53 found in other resistant clinical isolates (Fig 5A) [28,29,30].

To test if SrbA directly interacts with the predicted SREs, we assessed binding of recombinant SrbA to DNA-duplexes containing either SRE1, SRE2 or both sites by SPR (Fig 5B). SrbA showed interaction with both of the predicted binding sites within the 34 *mer*, however affinity for SRE1 (K_D_ = 22.8 nM) was 12-fold higher than that for SRE2 (K_D_ = 272.6 nM) (Fig 5B; panels 1 and 2 respectively). Employing a DNA duplex containing both SREs we measured a 2-fold increase in the saturating SrbA response (R_max_ value of 197.6) combined with an apparent K_D_ of 22.0 nM, which does not represent the simple average of the SrbA affinities measured for the single SREs. In conclusion, we propose cooperative binding of two SrbA homodimers to the 34 *mer* (Fig 5B; panel 3). Notably, the affinities of SrbA and the CBC to their partially overlapping binding motifs are at a similar level (74.4 vs. 22.0 nM), indicating that both regulators bind competitively to the 34 *mer* within the wt *cyp51A* promoter. By contrast, the 8-fold lower affinity for CBC at the CGAAT motif duplicated in TR34 will favor cooperative SrbA binding to both of the SREs in the 5´-TR34 region.

This finding suggests that the increased expression of *cyp51A* in strains harboring TR34, TR46 or TR53 could be the result of increased SrbA activity as a result of the duplication of its consensus binding sites and a lack of CBC and accordingly CBC/HapX repression.

### The 34 mer is crucial for activation of cyp51A gene expression and azole resistance

**Fig 6:**
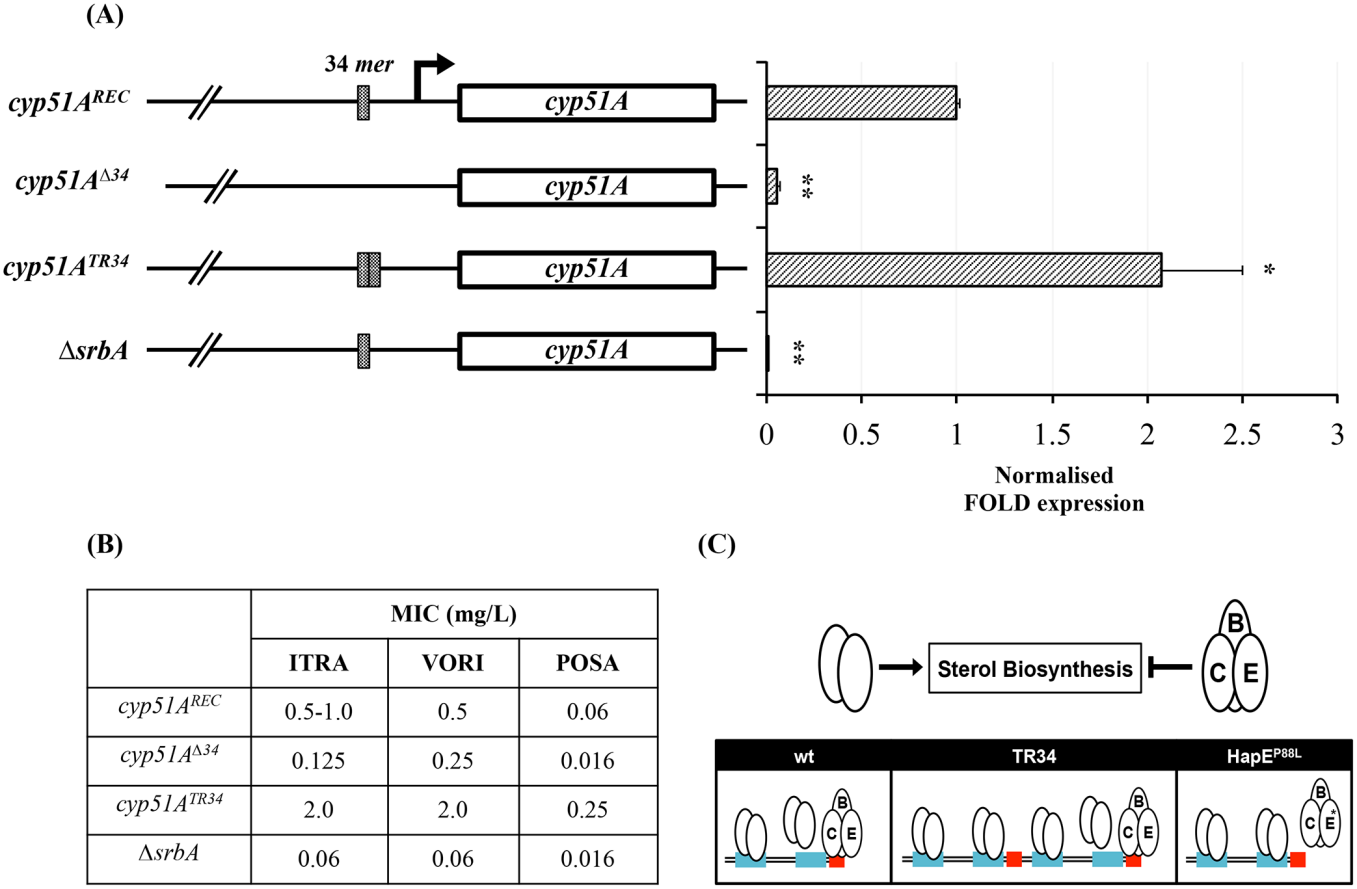
The 34 *mer* is essential for activation of *cyp51A* gene expression and azole resistance. (A) *cyp51A* transcript levels in strains lacking the 34 *mer* (*cyp51A* ^Δ*34*^) and strains carrying TR34 (*cyp51A* ^*TR34*^) have been measured using RT-qPCR (*gpdA* was used as reference gene). Strains for the expression analysis have been grown in AMM for 18h at 37°C, 200 rpm. Samples have been assessed in biological duplicates. Error bars indicate the standard deviation of respective samples. p-values were calculated by Student’s T-test (reference: *cyp51A* ^*REC*^): *, <0.05; ** <0.01; (B) MIC levels of itraconazole (ITRA), voriconazole (VORI) and posaconazole (POSA); (C) Proposed model highlighting the mechanistic basis of the 34 *mer* associated azole resistance in the promoter of *cyp51A*. In susceptible isolates (wt) SrbA homodimers bind to SRE1 and SRE2 thereby activating expression of *cyp51A*. The CBC competes with SrbA binding to the 34 *mer*. In resistant isolates, carrying the duplicated 34 *mer* (TR34), both SREs are duplicated whereas only the CGAAT motif located at the 3’ end of the 34 *mer* is effectively bound by the CBC. Upregulation of *cyp51A* in resistant isolates expressing mutated *hapE* (*hapE* ^*P88L*^) is caused by decreased binding affinity of the mutated complex. Hence, CBC-based competition with SrbA is perturbed enhancing binding of SrbA to the 34 *mer*.

Based on the hypothesis that the 34 *mer* is required for SrbA mediated *cyp51A* activation and consequent azole resistance, we generated a *cyp51A* promoter variant lacking the 34 *mer* (*cyp51A* ^Δ*34*^, S1 Fig). In order to confirm the previous results described by Snelders in 2011 [7] and to draw a direct comparison to the *cyp51A* ^Δ*34*^ isolate in an isogenic background, we also generated a strain carrying the tandem repeat TR34 (*cyp51A* ^*TR34*^). Deletion of the 34 *mer* (*cyp51A* ^Δ*34*^ *)* led to a >90% reduction in *cyp51A* expression similar to the *srbA* deletion mutant (Δ*srbA*) (Fig 6), which is consistent with our hypothesis. A similar result has been observed in a previous study using a *cyp51A* promoter based luciferase reporter approach [31]. In addition, the *cyp51A* ^Δ*34*^ isolate displayed a 4-fold reduction in MIC (from 0.50 mg/L to 0.13 mg/L, Fig 6), which mimics the azole susceptibility phenotype of the isogenic *cyp51A* deletion mutant (Δ*cyp51A*). MIC levels for the azoles were even lower for Δ*srbA*, which is likely a result of lack of activation of several ergosterol biosynthetic genes in this mutant [12,27].

Taken together, our data demonstrates that *cyp51A* activation relies on the presence of the 34 *mer*. This region is required for SrbA mediated activation of *cyp51A* gene expression, hence, for azole resistance in *A*. *fumigatus*.

## Discussion

In this study, we report a novel mechanism for the regulation of ergosterol metabolism in *A*. *fumigatus* and highlight the interplay between the transcriptional regulators SrbA and CBC. We also describe how perturbation of this regulatory mechanism through numerous routes can induce resistance to the primary class of agent used to treat fungal disease, the triazoles, which act by inhibiting ergosterol biosynthesis through depleting sterol C14-demethylase activity.

In *A*. *fumigatus*, the sterol regulatory element binding protein SrbA has been shown to be a positive acting transcriptional regulator of sterol biosynthesis and, aligned with this, has a crucial role in azole tolerance [14]. A recent study revealed that several ergosterol biosynthetic genes, including those encoding the two paralogs of Cyp51 (Cyp51A and Cyp51B) and Erg25 (Erg25A and Erg25B), are under direct control of SrbA [27], and strains lacking the regulator have significant reduction in the expression of the aforementioned genes. The role of SREBPs in activating expression of sterol biosynthesis is highly conserved.

In mammals, two genes encode three distinct isoforms SREBP-1a, SREBP-1c and SREBP-2, which are involved in positively regulating various aspects of lipid and sterol metabolism [10]. The activating role of the SREBPs in mammalian cells is facilitated by the action of the CCAAT-binding complex NF-Y.

The discovery that an amino acid alteration P88L within the HapE subunit of the CBC in *A*. *fumigatus* leads to resistance to all clinically used azole antifungals provided a potential link between CBC function and ergosterol biosynthesis and suggested the role of the CBC may also be conserved. Indeed in keeping with a role of the CBC in regulation of ergosterol biosynthesis we have demonstrated that the CBC binds the promoters of several genes in the pathway including *cyp51A* and *cyp51B*, *erg13B* and *erg7B*. Intriguingly, we identified that the CBC binds within the 34 *mer* in the promoter of *cyp51A* commonly duplicated in strains that are pan-azole resistant (TR34 family strains).

If the CBC were a positive regulator of ergosterol biosynthesis this may explain the mechanistic link between regulation of *cyp51A* and TR34 associated resistance. Our findings however are contrary to this hypothesis and suggest that the CBC of *A*. *fumigatus* is playing the opposite role to that of the mammalian orthologue and acting as a negative regulator of sterol biosynthesis. Lack of CBC activity (Δ*hapC*) leads to derepression of genes in the ergosterol biosynthetic pathway, including genes coding for enzymes of the initial and committed steps of sterol biosynthesis, *erg13A*, *erg13B* and *hmg1*, as well as the azole drug target *cyp51A* and a concomitant increase in sterol production and tolerance to drugs inhibiting the ergosterol biosynthetic pathway (azoles, allylamines and statins). Additionally, we have shown that the CBC binds efficiently only to the original CGAAT motif (Fig 2, CGAAT^-293^) but not to the duplicated version within TR34 (Fig 2, CGAAT^-327^). The reason for the differential binding affinities at these two sites were not investigated further however several lines of evidence point towards the importance of the bases outside this core motif as being critical. First, the structure of the *Aspergillus nidulans* CBC in complex with DNA demonstrated that hydrogen bonds between the phosphate groups of the DNA backbone outside the core motif, and the protein main or side chain atoms of HapC and HapE, significantly stabilise the protein:DNA complex and induce DNA bending. Second, alignment of the 23 bp *cycA* promoter sequence from which this structure was derived with the native and repeat *cyp51A* promoter sequences revealed different nucleotides at positions at which HapE amino acid residues mediate DNA sequence-independent interactions with phosphate oxygen atoms (S2 Fig). Finally our SPR data clearly indicate that an 8-fold lower CBC affinity to the duplicated TR34 CBC site is due to the presence of eight different nucleotides at the 3´end of the motif. Therefore, our working hypothesis is that altered DNA-bending might be the major cause for the decreased CBC affinity.

We have determined that the azole resistance exhibited by isolates with the HapE^P88L^ modification is linked to an inability of the modified CBC to bind effectively to its recognition site in the *cyp51A* promoter, leading to increased expression. Interestingly, the growth phenotype exhibited by a strain with the HapE^P88L^ mutation is less severe than that of the HapE null, suggesting only a partial loss of function. One could hypothesise two potential mechanisms for this partial phenotype, 1) the modified CBC retains binding capability for all targets but with reduced efficacy or 2) the modified CBC loses the ability to bind a subset of its targets. These scenarios are not mutually exclusive and more likely than not, a combination of both of these factors are responsible. Interestingly, structural analysis of the closely related *A*. *nidulans* CBC indicates that HapE stabilises the protein:DNA complex by binding through residues immediately adjacent to P88, namely L89 to K94 [23]. These residues are thought to interact in a DNA sequence-independent manner. Therefore it is likely that the modified CBC exhibits a general reduction in binding capability across all of its targets, with other factors such as the strength of binding of the CBC to the remaining sections of its binding site, or the presence of stabilising protein interaction partners dictating the strength of binding of the modified CBC.

Our genome-wide binding data revealed that the CBC interacts with the *cyp51A* promoter during sterol depletion as well as repletion. We found this to be true for almost all the ergosterol biosynthetic genes tested, which indicates that the complex might constitutively bind to these targets. This could suggest a simplistic binary regulatory mechanism at the promoters of sterol regulatory genes with the CBC carrying out a repressing role in the absence of an activator, *e*.*g*. SrbA. The regulation of sterol metabolism is however far more complex. We have demonstrated that the regulatory element HapX also has a role in the regulation of *cyp51A* and azole tolerance. As HapX is known to govern the regulation of genes involved in iron acquisition as well as iron consumption its association with *cyp51A*, a heme-iron containing enzyme is logical. We propose that HapX enhances or stabilises binding of the CBC at the *cyp51A* promoter in iron limiting conditions and by doing so provides a more nuanced way of regulating ergosterol biosynthesis in environments that are unfavorable for sterol production. It is possible therefore that further as yet undefined factors will have a role in the regulation of ergosterol biosynthesis.

Our data suggests a role for the CBC in TR34 family mediated azole resistance as its repressing function is not duplicated effectively in the repeat. This alone however, could not explain an increase in *cyp51A* expression observed in TR strains. Our hypothesis was that a positive regulator must be binding here. We therefore re-evaluated the sequence of the tandem repeat and identified two sequences that matched the consensus of the positive regulator SrbA and which were close to a putative binding site highlighted in a recent ChIP-seq study [27]. We confirmed SrbA interaction to both of these sequences in the 34 *mer* and cooperative binding at a region on the TR34. This leads to the conclusion that TR34 family driven upregulation of *cyp51A* is a result of effective duplication of SrbA binding sites in combination with ineffective duplication of the CBC site. It is interesting to note that tandem repeats of transcriptional enhancer elements are known mechanisms of azole resistance in several phytopathogenic moulds [32]. For example Hamamoto *et al*. identified a 126-bp tandem repeat in the *cyp51* 5’ NTR of the plant pathogen *Penicillium digitatum*, which caused resistance to Cyp51 inhibitors [33]. Whether resistance in this case is linked to duplication of SREs and/or ineffective duplication of repressor elements is unclear, however we have identified sequences within this repeat that resemble the SrbA binding consensus defined in *A*. *fumigatus* [27].

In contrast to *A*. *fumigatus* and most other eukaryotes, yeasts such as *Saccharomyces cerevisiae* and *Candida albicans* SREBPs have been replaced by Upc2p, a zinc-finger transcription factor which positively regulates sterol synthesis in these species [34]. In *C*. *albicans* antifungal azole resistance caused by overexpression of target genes has been extensively studied identifying Upc2p gain-of-function mutations. These types of mutations result in upregulation of the Cyp51 encoding gene and appear to be frequent in azole resistant *C*. *albicans* clinical isolates [35]. In *A*. *fumigatus* similar mutations in SrbA have not been identified so far. However, our study demonstrates an alternative route to enhance the activity of the positive regulator, SrbA, through increasing its DNA-binding ratio by duplicating its binding site.

The cause of the growth deficiency in the CBC null mutants is difficult to pinpoint exactly. The CBC is a global regulator, its consensus site is present in >30% eukaryotic promoters [22]. In *A*. *nidulans* and *A*. *fumigatus* several iron metabolic genes are known to be under control of the CBC in complex with HapX [19,24,36]. Additionally the CBC has also been shown to play a prominent role in the regulation of secondary metabolism, development and oxidative stress response [37,38]. These roles and probably the involvement of the CBC in many other biological processes are likely to be the cause for reduced fitness in the mutants. Although the same could be said for the role of the CBC in virulence, its interconnection with the transcription factor HapX is probably more important in this context. HapX mediated transcriptional adaptation to low iron environments has been demonstrated to be crucial for virulence in *A*. *fumigatus* and other pathogenic fungi such as *C*. *albicans*, *Cryptococcus neoformans* and *Fusarium oxysporum* [36,39,40,41]. Hence, we consider one major reason of the reduced pathogenicity to be a direct result of inefficient adaptation to low iron in the CBC mutant. It is interesting to note that despite a clear attenuation in virulence, an azole resistant CBC loss of function mutant, namely HapE^P88L^, was the likely cause of death in a chronic granulomatous disease patient [42].

This work provides novel insight into the molecular basis of sterol regulation and TR34 mediated azole drug resistance in *A*. *fumigatus* and suggests that if the binding of SrbA could be countered one could reverse drug resistance. The direct targeting of transcriptional binding is fraught with difficulties. Unlike many enzymes transcription factors have large surface areas for protein-protein and protein-DNA interactions which are difficult to disrupt with a small molecule. However modulation of these interactions may be possible using oligonucleotide or peptide therapeutics if these molecules can be effectively delivered into fungal cells [43]. Some, albeit limited, data suggests that this may be possible, at least in the case of oligonucleotides [44] and as significant differences exist between human SREBP and SrbA (less than 40% sequence identity at the DNA binding domain) selective targeting could be achieved. Alternatively, it should be possible to disrupt upstream activators of SrbA, for example the protease RbdA which was recently linked to SrbA cleavage and activation [45]. It is encouraging that a number of small molecules that inhibit Upc2-dependent transcriptional signaling *in vivo* have been identified in yeast, providing evidence that a similar, unbiased approach may be successful in *A*. *fumigatus* [46].

## Materials and Methods

### Ethics statement

All animal care procedures were supervised and approved by the Universitat Rovira i Virgili Animal Welfare and Ethics Committee.

### Strains, oligonucleotides and growth conditions

Strains and oligonucleotides used in this study are listed in S2 and S3 Tables. Conidia were grown on SAB agar plates. For phenotypic analysis, *A*. *fumigatus* liquid cultures were grown at 37°C in AMM according to Pontecorvo *et al*. [47]. AMM included 1% glucose as carbon source (w/v) and 20 mM ammonium tartrate as nitrogen source. *In vitro* susceptibility testing of mutant strains was carried out using the EUCAST broth microdilution reference method [26]. Strains are defined in this manuscript as resistant where their MIC exceeds the relevant breakpoint as defined by EUCAST [8].

### Generation of hapB, hapC, hapE, srbA, cyp51A deletion mutants and a strain expressing hapE^P88L^ in the CEA10 derivative A1160 Δku80 pyrG+ (A1160P+)

Coding sequence of *hapB*, *hapC*, *hapE*, *srbA* and *cyp51A* was disrupted in A1160P+. The deletion fragments for homologous recombination were generated using the FusionPCR approach previously described [48]. Around 1 kb of 5’ NTR and 1kb 3’ NTR were PCR amplified and subsequently linked to an antibiotic resistance cassette via PCR Fusion using primers listed in S3 Table. The P88L causing mutation of HapE was carried out using a similar FusionPCR strategy. *hapE* coding sequence and 1kb 3’ NTR were amplified from wt genomic DNA. The respective amplicons were linked to a hygromycin resistance conferring cassette via FusionPCR using a mutation generating forward primer.

### Reconstitution of Δcyp51A, ΔhapC and gfp-tagging of hapC

To complement Δ*cyp51A* and generate strains harboring modified *cyp51A* promoters, the basic plasmid pcyp51A^REC^, comprising a pyrithiamine resistance cassette, was generated. The plasmid for the reconstitution of Δ*hapC*, phapC^REC^, was generated following the same principle described for pcyp51A^REC^. The backbone of the hygromycin resistance cassette carrying plasmid pAN7-1 was amplified using primers pAN7-1-hapC-f/pAN7-1-hapC-r. *hapC* coding sequence plus 1.3 5’ NTR and 0.7 3’ NTR were amplified with primers hapCrec-f/hapCrec-r. Both fragments were gel-purified and linked via Gibson assembly as described above.

To tag *hapC* coding sequence at its 3’ end with *gfp*, phapC^REC^ was PCR amplified using primers phapC-GFP-FW/phapC-GFP-RV. The *gfp* encoding gene was amplified employing primers GFPhapC-FW/GFPhapC-RV using the recently described plasmid pgfpcccA as template [49]. Gel-purified fragments were combined using Gibson assembly yielding phapC^GFP^.

### Generation of cyp51A promoter mutants

Mutation of specific DNA sections was carried out according to the PCR based Q5 site-directed mutagenesis protocol (NEB). pcyp51A^REC^ was used as template DNA. TR34 was introduced into the promoter region employing primers TR34-FW/TR34-RV. Δ34-FW/Δ34-RV were used to delete the *34 mer*. After amplification of each construct yielding linearized PCR amplicons, PCR fragments were gel-purified and circularised using the Quick Ligation Kit (NEB). Plasmids carrying *cyp51A* promoter mutations were designated pcyp51A^TR34^ and pcyp51A^Δ34^.

### Fungal transformation

Generally 2 μg of the deletion constructs or 2 μg of the respective plasmids were used for transformation. Prior to transformation pcyp51A^REC^ and promoter mutated plasmids were linearised using *Bsr*GI, phapC^REC^ and phapC^GFP^ were digested with *Pml*I (S3 Fig). For transformation 1M sucrose was supplemented to AMM or SAB. Depending on the resistance cassette transformed, 0.1 mg/L pyrithiamine (*ptrA*), 0.2 g/L hygromycin B (*hph*) or 0.15 g/L zeocin (*ble*) were used for selection. AMM was used for pyrithiamine based transformation, SAB was used for hygromycin B and zeocin selection at pH 6 and pH 8, respectively.

### Sterol analysis: Cultivation and extraction of the cells

Liquid cultures for sterol measurements were grown in AMM for 24 h at 37°C, 200 rpm. Mycelia were harvested through filtration, shock frozen using liquid nitrogen and freeze-dried. The lyophilisate was ground and dissolved in 2M NaOH to obtain a suspension of 3.0 mg/mL. The work-up procedure can be taken from Müller *et al*. [50]. The residue was dissolved in 800 μL M*t*BE, 100 μL cholesterol solution (calibration standard, 10 μg/L), and 100 μL of silylation reagent MSTFA/TSIM (9:1) was added. The sample was gently shaken and stored for complete silylation at room temperature for at least 30 min, before being subjected to GC-IT-MS analysis [51,52].

Each sample was prepared in triplicate and measured in duplicate.

### Sterol analysis: GC-IT-MS

Sterols were analysed as trimethylsilyl (TMS) ethers. The sterol TMS ethers were identified by mass spectra and relative retention times (RRT) according to Alcazar-Fuoli *et al*. and Müller *et al*. [50,53]. The sterol TMS ether peaks were referred to the TMS ether peak area of the base peak of cholesterol TMS ether. The base peaks of each sterol TMS ether were taken as a quantifier ions for calculating the peak areas for cholestane *m/*z 217, cholesterol *m/z* 368, ergosta-5,7,9(11),22-tetraen-3β-ol (**1**) *m/z* 251, ergosterol (**2**) *m/z* 363, 5,6-dihydroergosterol (**3**) *m/z* 343, ergosta-5,7,24(28)-trien-3β-ol (**4**) *m/z* 363, episterol (**5**) *m/z* 343, lanosterol (**6**) *m/z* 393, eburicol (**7**) *m/z* 407, and 4,4-dimethylergosta-8,24(28)-dien-3β-ol (**8**) *m/z* 408. The content for each sterol [μg/mg] was calculated according to Müller and Bracher [52].

### Expression analysis

RNA was isolated using TRI Reagent (Sigma). 10 μg extracted total RNA were digested using RQ1 RNase-Free DNase (Promega) and further purified using the RNeasy Mini Kit (Qiagen). qPCR was performed in a 7500 Fast Real-Time PCR System (Applied Biosystems) using the iScript One-Step RT-PCR kit with SYBR Green (Cat# 170–8893)(Bio-Rad). Primers used for RT-qPCR analysis are listed in S3 Table. Amplification reactions were performed in a final volume of 25 μL using (1.0μL) 0.4μM forward primer, (1.0μL) 0.4 μM reverse primer, and 5 ng (5 μL) of total RNA.

### Bacterial expression and purification of proteins for SPR analysis

The *A*. *fumigatus* CBC consisting of HapB(230–299), HapC(40–137) and HapE(47–164) as well as HapX(24–158) were produced and purified as described by Gsaller *et al*. [19]. A HapE(47–164) subunit carrying the clinically relevant P88L mutation was generated employing a synthetic gene. The heterotrimeric HapB/HapC/HapE^P88L^ complex was purified to homogeneity by subsequent cobalt chelate affinity and size exclusion chromatography (SEC) just as the wt CBC (S4A Fig). The heterotrimeric status of the HapB/HapC/HapE^P88L^ complex, or in other words, its stability was proven by analytical SEC coupled light scattering measurements (S4B Fig). Therefore, an Äkta Explorer system (GE Healthcare) was connected to a miniDawn TREOS static light scattering (SLS) detector equipped with an internal quasi-elastic light scattering (QELS) system in series with an OPTILab T-rEX differential refractometer (Wyatt). Absolute molar mass and hydrodynamic radius (R_h_) were determined using the ASTRA 6 software (Wyatt). The CBC and CBC^P88L^ were chromatographed in 20 mM Tris/HCl, 400 mM NaCl, 1 mM DTT pH 7.5 using a Superdex 200 Increase 10/300 GL column (GE Healthcare).

The basic region/helix-loop-helix/leucine zipper (bHLHZ) region of *A*. *fumigatus* SrbA (amino acids 161–267) was purified following the procedure described in Linde *et al*. [54]. Real-time SPR protein-DNA interaction measurements were performed by using protocols published previously [19,54].

### Fluorescence microscopy

Microscopy images were taken on the Leica TCS SP8 inverted confocal microscope (Leica Microsystems CMS, Mannheim, Germany) using a 63×/1.2 NA objective. Images were captured with LAS AF V3.3. Images were processed using ImageJ and Adobe Photoshop CS6.

### Chromatin immunoprecipitation and ChIP-qPCR analysis

For ChIP-qPCR analysis 1x10^6^ spores/ml of *hapC* ^*GFP*^ were grown in 50 mL of AMM for 18 h at 37°C and 200 rpm.

ChIP was performed as previously described by Chung *et al*. [27] in this work using an Anti-GFP antibody (A-11122, Life technologies) or an anti-IgG control (ab46540, abcam) on Dynabeads Protein A magnetic beads (Thermo Fischer Scientific). Immunoprecipitated DNA was reverse cross-linked, treated with RNase A (Sigma), and then purified using a PCR purification kit (QIAGEN). ChIP’d DNA was eluted with 50 μL of elution buffer and 1 μL of the elution was used for ChIP-qPCR. All ChIP experiments were performed in biological duplicates.

ChIP-qPCR was carried out in an Applied Biosystems 7500 Fast Real-Time PCR System (Thermo Fischer Scientific). Amplification reactions were performed in a final volume of 20 μL using the iTaq Universal SYBR Green Supermix (Bio-Rad) with 0.4 μM forward primer, 0.4 μM reverse primer, and 1 μL of immunoprecipitated DNA. Oligonucleotides used in ChIP-qPCR are listed in S3 Table. Percent input method was applied to analyse the enrichment of the promoter region for each gene and the values were calculated according to the Thermo Fischer Scientific web site (https://www.thermofisher.com/uk/en/home/life-science/epigenetics-noncoding-rna-research/chromatin-remodeling/chromatin-immunoprecipitation-chip/chip-analysis.html). ChIP-qPCR experiments were run in triplicates and the results are presented together with the background signal and standard error.

### Virulence study in a murine model of systemic and pulmonary infection

Virulence of wt, Δ*hapC* and *hapC* ^*REC*^ was compared in a model of systemic and pulmonary infection, both developed in four-week old OF-1 male mice (Charles River; Criffa SA, Barcelona). For the pulmonary model, groups of 20 animals (10 for survival and 10 for fungal burden studies) were immunosuppressed with 125 mg/kg of cortisone acetate, given intraperitoneally (i.p.), administered four days prior infection and then 3 days per week. Animals were anaesthetized by inhalatory sevofluorane and challenged by nasal instillation (i.n) with conidial suspensions of each strain containing 1x10^5^ CFU/animal in a volume of 25 μl. Systemic infection was performed in groups of 16 animals (8 for survival and 8 for tissue burden studies) by intravenous (i.v.) inoculation into the lateral tail vein of 3x10^4^ CFU/animal of each strain. Five days after i.v. or i.n. infection, animals included in the tissue burden study were euthanatised by CO_2_ anoxia. Liver, lungs, kidneys, spleen and brain from animals challenged i.v. were aseptically removed for CFU determination, while lungs and kidneys were used in the pulmonary model. Approximately, one half of each organ was weighted and mechanically homogenized in 1 to 1.5 mL of PBS. Homogenates were serially 10-fold diluted and placed onto potato dextrose agar plates for CFU/g determination.

## Supporting Information

S1 Fig Effects of *hapC* deletion on fungal load in organs of immunosuppressed mice. (A) Cortisone acetate immunosuppressed OF-1 mice challenged intranasally with 1x10^5^ CFU/animal of *A*. *fumigatus*. Horizontal bars represent the median. ^a^P < 0.05 vs. wt, ^b^P < 0.05 vs. *hapC* ^*REC*^; (B) Cyclophosphamide immunosuppressed OF-1 mice challenged intravenously with 3x10^4^ CFU/animal of *A*. *fumigatus*. Horizontal bars represents the median. ^a^P < 0.05 vs. wt, ^b^P < 0.05 vs. *hapC* ^*REC*^.
(TIF)

S2 Fig Alignment of the native (-293) and repeat region (-327TR) of the *cyp51A* promoter bound by the CBC with the *A*. *nidulans cycA* region whose structure was elucidated in complex with the AnCBC. Yellow highlights indicate bases conserved in all three binding sites, blue in two of the three. Underlined bases identify those outside the core domain shown to be linked with the CBC.
(TIF)

S3 Fig Scheme of site-directed integration of plasmids for *cyp51A* promoter mutations and *gfp*-tagged *hapC*. (A) *cyp51A* based plasmids were linearised using *Bsr*GI and transformed into Δ*cyp51A*. The same transformation procedure was carried out for all plasmids containing modified *cyp51A* promoter versions. (B) Site-directed integration of a plasmid harboring C-terminally tagged *hapC* into Δ*hapC*.
(TIF)

S4 Fig Characterisation of purified recombinant proteins used in this study. (A) SDS-PAGE analysis of wt (HapB/HapC/HapE) and mutated CBC (HapB/HapC/HapE^P88L^) as well as recombinant HapX(24–158) protein. (B) Analysis of the solution heterotrimeric state of HapB/C/E and HapB/C/E^P88L^ complexes by analytical size exclusion chromatography coupled light scattering measurements. The static light scattering (SLS) signals are shown overlaid with the calculated absolute molar masses across the elution profiles. Determined molar masses (M_w_) and hydrodynamic radii (R_h_) are plotted inside the graph. Note that HapB/C/E^P88L^ elutes slightly later than HapB/C/E, which fits with a lower R_h_ value and indicates a more compact solution structure of the HapB/C/E^P88L^ complex.
(TIF)

S1 Table ChIP-Seq Peaks from genes coding for enzymes of the ergosterol biosynthetic pathway. Cultures were grown for 18 h in AMM at 37°C. TSS, putative transcriptional start site.
(DOCX)

S2 Table Strains used in this study. (DOCX)

S3 Table Oligonucleotides used in this study (5´→3´). (DOCX)
