# Correction: Sterol Biosynthesis and Azole Tolerance Is Governed by the Opposing Actions of SrbA and the CCAAT Binding Complex

**DOI:** 10.1371/journal.ppat.1006106

**Published:** 2016-12-14

**Authors:** Fabio Gsaller, Peter Hortschansky, Takanori Furukawa, Paul D. Carr, Bharat Rash, Javier Capilla, Christoph Müller, Franz Bracher, Paul Bowyer, Hubertus Haas, Axel A. Brakhage, Michael J. Bromley

The following information is missing from the Funding section: This work has also received funding from the Medical Research Council (MR/M02010X/1) to MJB. The funders had no role in the in the study design, data collection and analysis, decision to publish, or preparation of the manuscript.

## References

[1] Gsaller F, Hortschansky P, Furukawa T, Carr PD, Rash B, Capilla J, et al (2016) Sterol Biosynthesis and Azole Tolerance Is Governed by the Opposing Actions of SrbA and the CCAAT Binding Complex. PLoS Pathog 12(7): e1005775 doi: 10.1371/journal.ppat.1005775 27438727 10.1371/journal.ppat.1005775 PMC4954732

