# Supplementary figures and images for: Sterol Biosynthesis and Azole Tolerance Is Governed by the Opposing Actions of SrbA and the CCAAT Binding Complex
Source: PLoS Pathog. 2016 Jul 20;12(7):e1005775. doi: 10.1371/journal.ppat.1005775 (PMC4954732; doi:10.1371/journal.ppat.1005775)

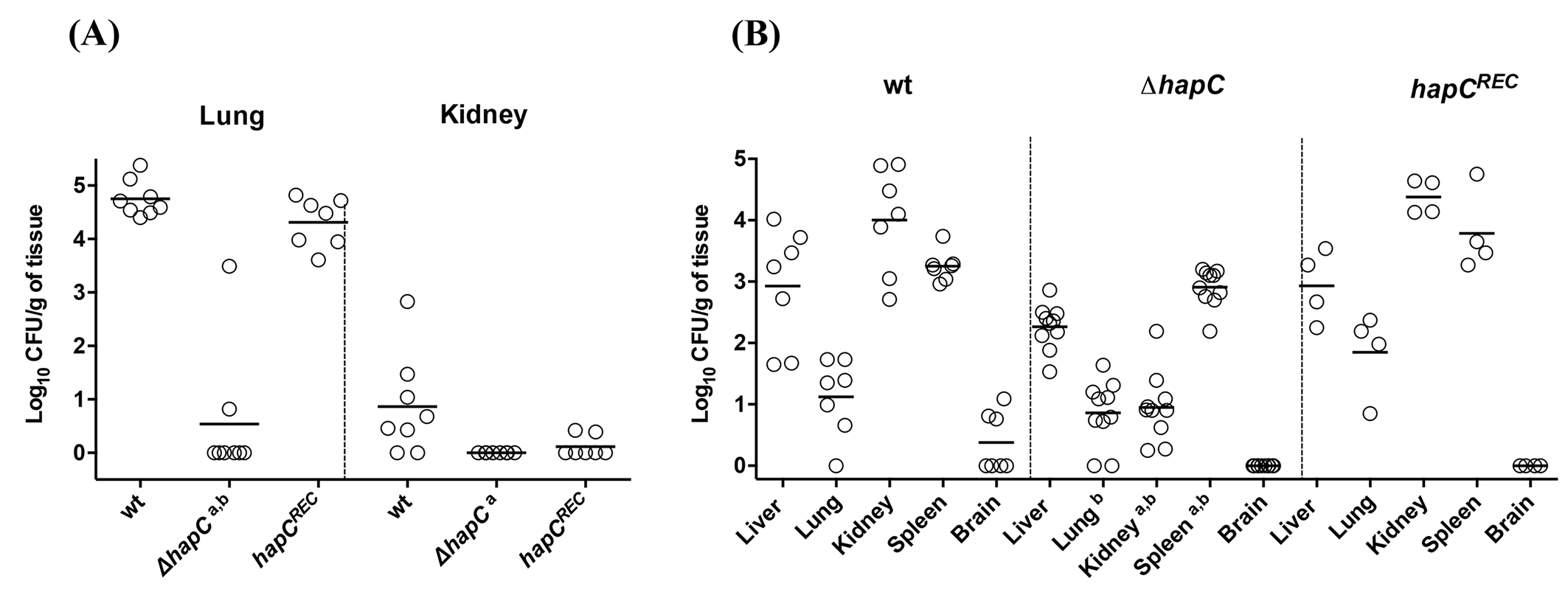

Supplement: S1 Fig — (A) Cortisone acetate immunosuppressed OF-1 mice challenged intranasally with 1x10^5^ CFU/animal of A. fumigatus. Horizontal bars represent the median. aP < 0.05 vs. wt, bP < 0.05 vs. hapC REC; (B) Cyclophosphamide immunosuppressed OF-1 mice challenged intravenously with 3x10^4^ CFU/animal of A. fumigatus. Horizontal bars represents the median. aP < 0.05 vs. wt, bP < 0.05 vs. hapC REC. (TIF) [file ppat.1005775.s001.tif]

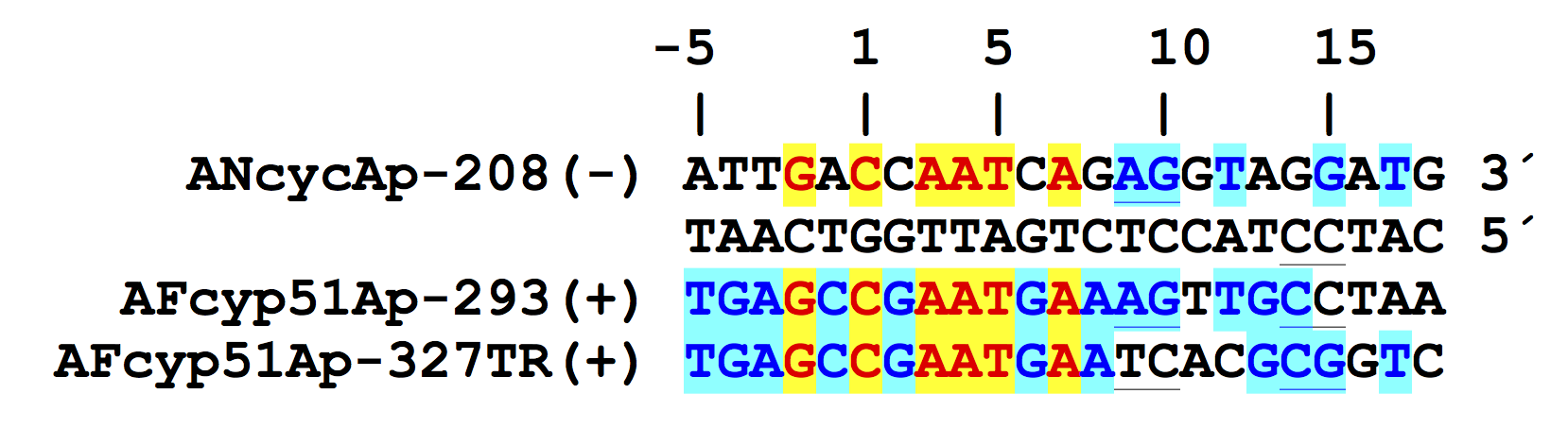

Supplement: S2 Fig — Yellow highlights indicate bases conserved in all three binding sites, blue in two of the three. Underlined bases identify those outside the core domain shown to be linked with the CBC. (TIF) [file ppat.1005775.s002.tif]

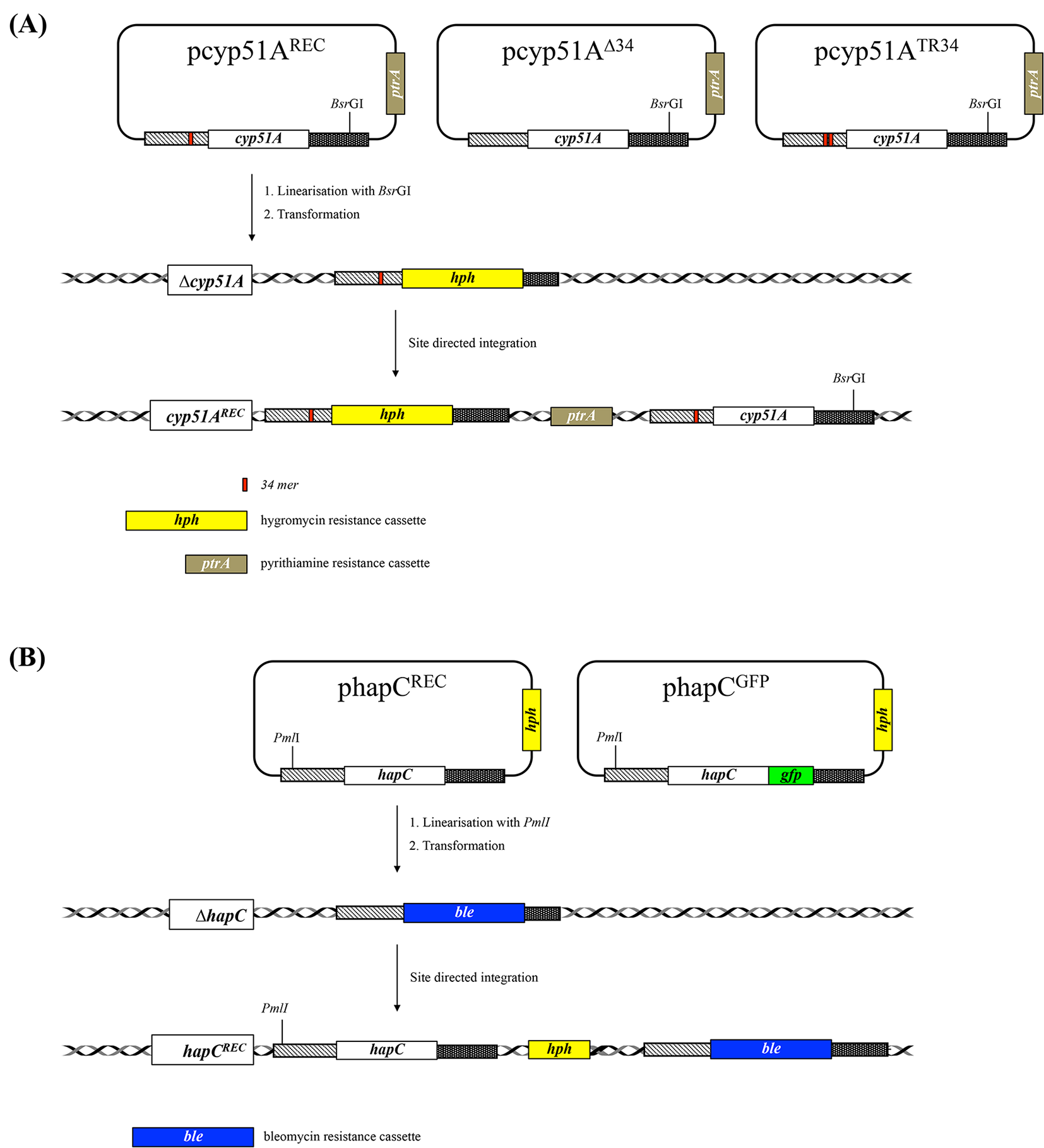

Supplement: S3 Fig — (A) cyp51A based plasmids were linearised using BsrGI and transformed into Δcyp51A. The same transformation procedure was carried out for all plasmids containing modified cyp51A promoter versions. (B) Site-directed integration of a plasmid harboring C-terminally tagged hapC into ΔhapC. (TIF) [file ppat.1005775.s003.tif]

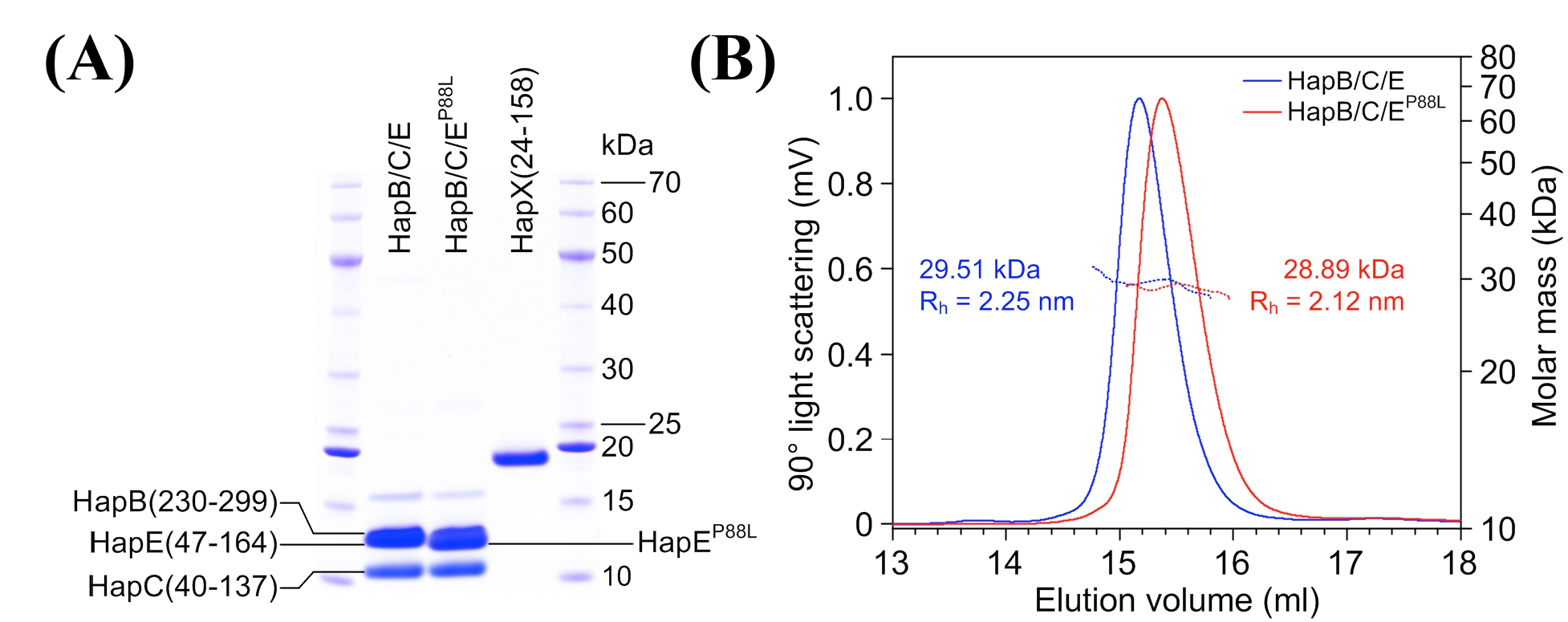

Supplement: S4 Fig — (A) SDS-PAGE analysis of wt (HapB/HapC/HapE) and mutated CBC (HapB/HapC/HapEP88L) as well as recombinant HapX(24–158) protein. (B) Analysis of the solution heterotrimeric state of HapB/C/E and HapB/C/EP88L complexes by analytical size exclusion chromatography coupled light scattering measurements. The static light scattering (SLS) signals are shown overlaid with the calculated absolute molar masses across the elution profiles. Determined molar masses (Mw) and hydrodynamic radii (Rh) are plotted inside the graph. Note that HapB/C/EP88L elutes slightly later than HapB/C/E, which fits with a lower Rh value and indicates a more compact solution structure of the HapB/C/EP88L complex. (TIF) [file ppat.1005775.s004.tif]
