# Supplementary material for: Sterol Biosynthesis and Azole Tolerance Is Governed by the Opposing Actions of SrbA and the CCAAT Binding Complex
Source: PLoS Pathog. 2016 Jul 20;12(7):e1005775. doi: 10.1371/journal.ppat.1005775 (PMC4954732; doi:10.1371/journal.ppat.1005775)
Supplement: S1 Table — Cultures were grown for 18 h in AMM at 37°C. TSS, putative transcriptional start site. (DOCX) [file ppat.1005775.s005.docx]

Table S1 **ChIP-Seq Peaks from genes coding for enzymes of the ergosterol biosynthetic pathway.** Cultures were grown for 18 h in AMM at 37°C. TSS, putative transcriptional start site.

| **Gene ID** | **Fold enrichment** | **Strand** | **Chromosome** | **Peak Summit** | **distance to TSS** | **TSS** | **Putative gene function** |
| --- | --- | --- | --- | --- | --- | --- | --- |
| AFUB_000550 | 3.34 | - | DS499594 | 152889 | 304 | 152585 | acetyl-CoA acetyltransferase |
| AFUB_038500 | 3.74 | - | DS499596 | 1310486 | 429 | 1310057 | HMG-CoA synthase |
| AFUB_020770 | 1.60 | + | DS499595 | 974403 | 1151 | 975554 | HMG-CoA reductase |
| AFUB_064220 | 2.8 | + | DS499598 | 398730 | 175 | 398905 | diphosphomevalonate decarboxylase |
| AFUB_087800 | 2.1 | + | DS499601 | 323597 | 280 | 323877 | farnesyl-diphosphate farnesyltransferase |
| AFUB_055310 | 5.88 | + | DS499597 | 1842270 | 285 | 1842555 | squalene monooxygenase |
| AFUB_069030 | 1.54 | - | DS499598 | 1727349 | 324 | 1727025 | lanosterol synthase |
| AFUB_063960 | 2.36 | - | DS499598 | 332820 | 297 | 332523 | sterol C14-demethylase |
| AFUB_089270 | 2.76 | - | DS499601 | 799943 | 358 | 799585 | sterol C14-demethylase |
| AFUB_098170 | 3.65 | - | DS499603 | 11835 | 316 | 11519 | sterol C4-methyl oxidase |
| AFUB_084150 | 3.36 | + | DS499600 | 1281799 | 263 | 1282062 | sterol C4-methyl oxidase, putative |
| AFUB_099400 | 3.65 | + | DS499603 | 346900 | 361 | 347261 | sterol C24-methyltransferase |
| AFUB_066290 | 3.67 | + | DS499598 | 955431 | 364 | 955795 | S-adenosyl-methionine-sterol-C-methyltransferase |
| AFUB_066290 | 4.93 | + | DS499598 | 954834 | 961 | 955795 |  |
| AFUB_004350 | 3.72 | + | DS499594 | 1212336 | 458 | 1212794 | sterol C22-desaturase |
