# Supplementary material for: Sterol Biosynthesis and Azole Tolerance Is Governed by the Opposing Actions of SrbA and the CCAAT Binding Complex
Source: PLoS Pathog. 2016 Jul 20;12(7):e1005775. doi: 10.1371/journal.ppat.1005775 (PMC4954732; doi:10.1371/journal.ppat.1005775)
Supplement: S2 Table — (DOCX) [file ppat.1005775.s006.docx]

| Strain | **Reference** |
| --- | --- |
| wt, A1160P+ | [[1](#_ENREF_1)] |
| ∆*hapB* | This study |
| ∆*hapC* | This study |
| ∆*hapE* | This study |
| ∆*srbA* | This study |
| *∆cyp51A* | This study |
| *∆hapC∆cyp51A* | This study |
| *cyp51A^REC^* | This study |
| *hapC^REC^* | This study |
| ∆*hapX* | This study |
|  |  |
| **P88L mutated HapE** |  |
| *hapE^P88L^* | This study |
|  |  |
| **Promoter mutants** |  |
| *cyp51A^∆34^* | This study |
| *cyp51A^TR34^* | This study |
|  |  |
| ***gfp*-tagged *hapC*** |  |
| *hapC^GFP^* | This study |
|  |  |

Table S2 **Strains used in this study.**

**REFERENCES**

1. Fraczek MG, Bromley M, Buied A, Moore CB, Rajendran R, et al. (2013) The cdr1B efflux transporter is associated with non-cyp51a-mediated itraconazole resistance in Aspergillus fumigatus. J Antimicrob Chemother 68: 1486-1496.
