# Supplementary material for: Sterol Biosynthesis and Azole Tolerance Is Governed by the Opposing Actions of SrbA and the CCAAT Binding Complex
Source: PLoS Pathog. 2016 Jul 20;12(7):e1005775. doi: 10.1371/journal.ppat.1005775 (PMC4954732; doi:10.1371/journal.ppat.1005775)
Supplement: S3 Table — (DOCX) [file ppat.1005775.s007.docx]

Table S3 **Oligonucleotides used in this study (5´**→**3´).**

| ***(A) Amplification of the resistance-conferring cassette.*** | |  |
| --- | --- | --- |
| hph-FW | CCGGCTCGGTAACAGAACTAACGGCGTAACCAAAAGTCAC |  |
| hph-RV | GGGAGCATATCGTTCAGAGCTCTTGACGACCGTTGATCTG |  |
| ***(B) hapB disruption.*** |  |  |
| hapB-1 | CGCATACATCCTTTAGCCGT |  |
| hapB-2RV | TAGTTCTGTTACCGAGCCGGTTCCGTCACCTTGGTACCTT |  |
| hapB-3 | GCTCTGAACGATATGCTCCAACTTTCGCCACTTCTTTCTCGT |  |
| hapB-4RV | TGCACTCAAGCCAGATATGC |  |
| hapB-N1 | GCATTATCATCCGAGACCGT |  |
| hapB-N2 | CACAGATGCATGTCCACCTC |  |
| ***(C) hapC disruption.*** |  |  |
| hapC-1 | CACAGATACGGATAGTCACG |  |
| hapC-2RV | TAGTTCTGTTACCGAGCCGGCGGATACTGGATAAGGTGTG |  |
| hapC-3 | GCTCTGAACGATATGCTCCCCAAGACTCTCACACACTCCA |  |
| hapC-4RV | ACTCCACGTCTCTACCACAG |  |
| hapC-N1 | ACTTTGTGTCTCAGCTCGTC |  |
| hapC-N2 | GATCAGCCTAGGGAATTTCT |  |
| ***(D) hapE disruption.*** |  |  |
| hapE-1FW | AATGAGGAGTCCGTTGCGAG |  |
| hapE-2RV | TAGTTCTGTTACCGAGCCGGAGTCAACAGAAGCGGCAAGT |  |
| hapEc-3FW | GCTCTGAACGATATGCTCCCAGATGGCTTGGTCAGTCAGC |  |
| hapEc-4RV | GCATCGACATCCGCAACTTC |  |
| hapE-N1 | TGCATATTCGTGGAAAGTGG |  |
| hapEc-N2 | CGCCAATGAAGATTTCTCGCA |  |
| ***(E) cyp51A disruption.*** |  |  |
| cyp51A-1 | GAGCTGATTTGCCATGGATT |  |
| cyp51A-2RV | TAGTTCTGTTACCGAGCCGGCCCGAGGATTAGAGTTGCAG |  |
| cyp51A-3 | GCTCTGAACGATATGCTCCCCCATCGAGGACTTCAAAGGA |  |
| cyp51A-4RV | AGTGGCAAACGGACTTTGAG |  |
| cyp51A-N1 | AACTGCACAAAAGGGTGTCC |  |
| cyp51A-N2 | GAAACTCCCATCAGGTCGTG |  |
| \| *(F) hapX disruption.* \| \| \| \| --- \| --- \| --- \| \| hapX-1FW \| TAAGTAGTTGCTGTGCGCGT \| \| hapX-2RV \| TAGTTCTGTTACCGAGCCGGCTGGATGGACATTAGTGGGG \| \| hapX-3FW \| GCTCTGAACGATATGCTCCAACATGATACCCAAACGGACTCG \| \| hapX-4RV \| ATTGGTATGTGGGGTATCGC \| \| hapX-N1 \| TACGGATTGCGTTGCTGTAG \| \| hapX-N2 \| GGAGGGGATGAGGAAGTGAT \|  \| *(G) srbA disruption.* \| \| \| \| --- \| --- \| --- \| \| srbA-1FW \| GTCGCGGAGTATCCCAAATA \| \| srbA -2RV \| TAGTTCTGTTACCGAGCCGGTGTTTCACTGCTGGTCTTGG \| \| srbA -3FW \| GCTCTGAACGATATGCTCCAACAAAGCTGGCGAGCAGAGTAG \| \| srbA -4RV \| AAGGCCTAGACAACACGCAG \| \| srbA -N1 \| CGCGTTATCTCCAGGTTGAT \| \| srbA -N2 \| CGTCAGGGAAGAGCAAAGAC \|   ***(H) Generation of phapC^REC^*.** | |  |
| hapCrec-f | GGTTTAAACGAATTCGCCCTTctcgcactactgtcactcc | |
| hapCrec-r | GGAGAAAATACCGCATCAGGCGCCAATATCAAAATCCGGG | |
| pAN7.1-hapC-f | CCTGATGCGGTATTTTCTCC | |
| pAN7.1-hapC-r | AAGGGCGAATTCGTTTAAACCtgcaGGTCGAGTGGAGATG | |
| *(I) Generation of phapC^GFP^.* | |  |
| GFPhapC-FW | ACTCGTACGATATCAGATCTGTGAGCAAGGGCGAGGAG |  |
| GFPhapC-RV | gatgcatTCACTTGTACAGCTCGTCCATGC |  |
| phapC-GFP-FW | GCTGTACAAGtgaatgcatcatgtgtgacaga |  |
| phapC-GFP-RV | AGATCTGATATCGTACGAGTCGCCTCCTGCTC |  |
| *(J) Generation of pcyp51A^REC^.* | | |
| cyp51A-FW | CGGCCGCTCTAGAACTAGTTCCACCCATCAACAGAAACA | |
| cyp51A-RV | CCTCTTTAGCTTTGACCGGTCCAGTTTGAACACGGAACCT | |
| pSK275-FW | ACCGGTCAAAGCTAAAGAGG | |
| pSK275-RV | ACTAGTTCTAGAGCGGCCG | |

| *(K) Generation of promoter mutants (TR34, ∆34).* | |
| --- | --- |
| TR34-FW | **P-**ATGTGTGCTGAGCCGAATGAATCACGCGGTCCGGATGTG |
| TR34-RV | CCGGACCGCGTGATTCTAGACAACTCTGAAGTGGTGCTGCG |
| ∆34-FW | **P-**GAAAGTTGCCTAATTACTAAGGTGT |
| ∆34-RV | TAGACAACTCTGAAGTGGTGCT |

**P-**, phosphate group.

***(L) Generation of hapE^P88L^.***

| hapEc-1FW | TATTCACAGGTCCCGCAACC |
| --- | --- |
| hapEc-2RV | TAGTTCTGTTACCGAGCCGGGAGCATGGCGCCGTTAAAAT |
| hapEc-3FW | GCTCTGAACGATATGCTCCCAGATGGCTTGGTCAGTCAGC |
| hapEc-4RV | GCATCGACATCCGCAACTTC |
| hapEP88L-N1 | CACCAATTGCTACTGGCCC |
| hapEc-N2 | CGCCAATGAAGATTTCTCGCA |

***(M) qRT-PCR based expression analysis.***

| RTerg13A-f | TCTATGATCAG*ATGTGTAAGCTCCG |
| --- | --- |
| RTerg13A-r | GCATCATCCACATGCACCAG |
| RTerg13B-f | AGACTTATGATGAG*ATGTGCATCCT |
| RTerg13B-r | TGTCGTCAATCTCGGTCAGG |
| RThmg1-f | CAGGACTACACG*GGCGTC |
| RThmg1-r | TTGAGCAGAGGCATTGGAAG |
| RThmg2-f | CTTCTTGGACCCA*GTTGCAT |
| RThmg2-r | CACTAGGTGTTCGAGAGACG |
| RTerg7B-f | AGGGCTTCCTCTG*GATCTCC |
| RTerg7B-r | CCCGGTGGAAGCTGTAGTTT |
| RTcyp51A-f | GTGCAGAGAAAAG*TATGGCG |
| RTcyp51A-r | TCCGCATTGACATCCTTGAG |
| RTcyp51B-f | GGAGCAGAAGAAG*TTCGTCA |
| RTcyp51B-r | GGAACGCCGGAGAATTTTTG |
| RTgpdA-FW | GAGCTCAAAA*ACATCCTCGGC |
| RTgpdA-RV | CGAAGTTGGGGTTGAGGGAG |

*EXON/INTRON junction.

***(N) ChIP-qPCR based in vivo DNA binding analysis.***

| ChIPactA-FW | CTGGGTTCGTAGTTCGGGTA |
| --- | --- |
| ChIPactA-RV | GGGGTTCACTCGGATGTTTA |
| ChIPerg13B-FW | TCTGCTGTGCCTTACCAAGA |
| ChIPerg13B-RV | GCGCTCTTCCGTAGCTAAAC |
| ChIPerg7B-FW | GGCAAGTGACGTCATCATCA |
| ChIPerg7B-RV | CCACCCCCTCTGCTTATATGT |
| ChIPcyp51A-FW | TAATCGCAGCACCACTTCAG |
| ChIPcyp51A-RV | TTCACCTACCTACCAATATAGGTTCA |
| ChIPcyp51B-FW | TGGATCCTTGGCGATACAAC |
| ChIPcyp51B-RV | CCCCTGGACTAACTGACCCTA |

***(O) SPR based in vitro DNA binding analysis.***

| Bcyp51Ap-293-37 | **B-**GCTGAGCCGAATGAAAGTTGCCTAATTACTAAGGTGT |
| --- | --- |
| cyp51Ap-293-37 | ACACCTTAGTAATTAGGCAACTTTCATTCGGCTCAGC |
| Bcyp51Ap-TR34-37 | **B-**GCTGAGCCGAATGAATCACGCGGTCCGGATGTGTGCT |
| cyp51Ap-TR34-37 | AGCACACATCCGGACCGCGTGATTCATTCGGCTCAGC |
| Bcyp51Ap-320-16 | **B-**AGAATCACGCGGTCCG |
| cyp51Ap-320-16 | CGGACCGCGTGATTCT |
| Bcyp51Ap-295-16 | **B-**CGGCTCAGCACACATC |
| cyp51Ap-295-16 | GATGTGTGCTGAGCCG |
| Bcyp51Ap-320-32 | **B**-AGAATCACGCGGTCCGGATGTGTGCTGAGCCG |
| cyp51Ap-320-32 | CGGCTCAGCACACATCCGGACCGCGTGATTCT |

**B-**, biotin group.
